# Factors influencing antiretroviral treatment suboptimal adherence among perinatally HIV-infected adolescents in Thailand

**DOI:** 10.1371/journal.pone.0172392

**Published:** 2017-02-16

**Authors:** Luyi Xu, Kerim Munir, Cheeraya Kanabkaew, Sophie Le Coeur

**Affiliations:** 1 Harvard Medical School, Boston, United States of America; 2 Developmental Medicine Center, Division of Developmental Medicine, Boston Children's Hospital, Boston, United States of America; 3 Institut de recherche pour le développement (IRD) 174-PHPT, Chiang Mai, Thailand; 4 Faculty of Associated Medical Sciences, Chiang Mai University, Chiang Mai, Thailand; 5 Institut National d’Etudes Démographiques (INED), Paris, France; 6 Harvard T.H. Chan School of Public Health, Boston, MA, United States of America; UCL Institute of Child Health, University College London, UNITED KINGDOM

## Abstract

**Background:**

Existing studies have suggested decreased adherence and rebound in mortality in perinatally HIV-infected adolescents receiving antiretroviral therapy (ART) as compared to adults and young children.

**Methods:**

We used both quantitative and qualitative approaches to identify factors influencing adherence among perinatally infected adolescents in Thailand. We analyzed data from 568 pairs of perinatally infected adolescents (aged 12–19) and their primary caregivers in the Teens Living With Antiretrovirals (TEEWA) study, a cross-sectional survey conducted in 2010–2012. We also conducted 12 in-depth interviews in 2014 with infected adolescents or their primary caregivers to elicit experiences of living with long-term ART.

**Results:**

From the quantitative analysis, a total of 275 (48.4%) adolescents had evidence of suboptimal adherence based on this composite outcome: adolescents self-reported missing doses in the past 7 days, caregiver rating of overall adherence as suboptimal, or latest HIV-RNA viral load ≥1000 copies/ml. In multivariate logistic regression analysis, younger age, having grandparents or extended family members as the primary caregiver, caregiver-assessed poor intellectual ability, having a boy/girlfriend, frequent online chatting, self-reported unhappiness and easiness in asking doctors questions were significantly associated with suboptimal adherence. From the in-depth interviews, tensed relationships with caregivers, forgetfulness due to busy schedules, and fear of disclosing HIV status to others, especially boy/girlfriends, were important contributors to suboptimal adherence. Social and emotional support and counseling from peer group was consistently reported as a strong adherence-promoting factor.

**Conclusion:**

Our findings highlight unique barriers of ART adherence among the perinatally infected adolescents. Future interventions should be targeted at helping adolescents to improve interpersonal relationships and build adaptive skills in recognizing and addressing challenging situations related to ART taking.

## Introduction

With the advances in effectiveness and availability of antiretroviral therapy (ART), perinatally infected children are now surviving to adolescence and emerging as a new group in the global HIV/AIDS pandemic. UNAIDS estimates that 2.0 million adolescents aged 10–19 were living with HIV in 2014[1], among which 9,600 were in Thailand[2]. Despite increasing access to ART, several studies in Thailand and worldwide have found decreased adherence and rebound in mortality in adolescents receiving ART as compared to adults and young children[3–5], with poorer adherence when children advance in age[6–8]. Good adherence to the ART is crucial for successful viral suppression, as incomplete adherence leads to an increase in HIV viremia, risk of treatment failure, and accumulating resistance mutations[9–11].

In Thailand, HIV services are delivered mostly through the National Health Security Office, and Thai nationals receive free ART treatment. Coverage of ART among children (aged 0–14) and adults (aged 15+) in Thailand were estimated to be 65% and 61% as of 2014, respectively[1]. After successful initiation of ART at tertiary (provincial) hospitals, children are often referred back to a district hospital for follow-ups[12]. At the time of the study, the first and second-line treatments were NRTI/NNRTI-based and PI-based, respectively. Integrase inhibitors were barely available[13]. For perinatally infected children and adolescents, disclosure of HIV status is recommended from age 7, through hospital-specific practices, after evaluating the child’s readiness[14]. In terms of psychosocial interventions, peer groups (frequently led by physicians), NGOs or volunteer networks organize home visitations, scholarship programs, life skills trainings and educational activities and camps to provide support and raise public awareness.

The perinatally infected adolescents have been increasingly recognized as a specific group with unique challenges in HIV/AIDS treatment[3, 15–17]. Compared to behaviorally infected peers, perinatally infected youth have significantly more barriers to ART adherence[18], including a more complicated clinical course, early-life adversities featuring loss of parent(s) and family instability, experiences of discrimination and trauma from disclosure of their HIV status[5, 7, 8, 19–21]. As they advance in age, they also face the changes from pediatric to adult care, and the critical transition from dependence on caregivers to assuming full responsibilities on their own, including the maintenance of ART adherence[12, 22].

There have been increasing concerns about the limited available data on ART adherence pertaining specifically to the perinatally infected adolescents, especially in resource-limited settings[15, 16, 23]. Systematic reviews suggested wide variations of factors influencing ART adherence among adolescents in different social and cultural environments[23, 24]. Moreover, studies on ART adherence using only quantitative analysis may result in incomplete understanding of the contextual issues in everyday life unique to the local area. To address these limitations, we conducted one of the very first studies in Thailand that used both quantitative and qualitative approaches and included perspectives from both the perinatally infected adolescents and their primary caregivers. We aim to achieve a comprehensive assessment of factors influencing ART adherence among this particular population to help guide targeted interventions in the future.

## Methods

Our study was comprised of a quantitative cross-sectional survey, the Teens Living With Antiretrovirals (TEEWA) study (2010–2012) and qualitative in-depth interviews (IDIs) with perinatally HIV-infected adolescents on ART and their primary caregivers (2014). Data analysis was performed after completion of all data collection; therefore, the design of the qualitative component was not influenced by the results of the quantitative data. The study was approved by the Ethics Committee, Faculty of Associate Medical Sciences, Chiang Mai University, and the Institutional Review Board of the Harvard University Faculty of Medicine. A written informed consent was obtained from the adolescent’s primary caregiver for their own and their adolescent’s interview; a separate written assent was obtained from the adolescents themselves. All adolescent participants were informed that their caregivers would be interviewed about them but responses from each party would be kept confidential from the other. All participants were assured that the information provided was kept confidential for research purpose only. All data collected were anonymised using unique study identifiers.

### Quantitative component

Quantitative data were derived from TEEWA, a cross-sectional survey conducted in 2010–2012 by the Program for HIV Prevention and Treatment (PHPT) to investigate the situations and needs of perinatally HIV-infected adolescents[25]. The study targeted all perinatally infected adolescents aged 12 to 19 receiving ART from 20 participating hospitals, covering rural, periurban, and urban areas throughout Thailand. None of the adolescents had been transferred to adult care yet.

This quantitative survey has two components. First, a self-administered questionnaire was filled by the adolescent, which documented aspects of everyday life including household environment, school, work, health/medications, interpersonal relationships, and daily activities. There was no reference to HIV, AIDS or ART in the adolescent’s questionnaire in order to prevent unintended disclosure. Adolescents were asked if they took any medications on a regular basis, without specifying the name or type of the medications. Second, a face-to-face guided survey of the adolescent’s primary caregiver was conducted by two trained interviewers—one psychiatric nurse and one social scientist. The caregiver’s survey collected information on socio-demographic characteristics of the caregiver, and major events in the adolescent’s life history including illness/death of biological parents, HIV-related medical history, ART treatment and adherence, disclosure of HIV status to the adolescent, caregiver-adolescent relationship, and experiences of discrimination. Additionally, adolescents’ clinical characteristics at HIV diagnosis, initiation of ART and latest CD4, viral load (VL) and ART status were also collected based on chart reviews.

This analysis focused on the predictors of suboptimal ART adherence, utilizing all available data from the TEEWA study: 1) adolescent’s self-reported missed doses in the past 7 days; 2) caregiver’s rating of overall adherence (very good, good, average, poor, very poor); and 3) laboratory records of latest VL. In this study, an adolescent was considered adherent if he/she meets all three of the following criteria: no self-reported missed dose in the past 7 days, receiving a rating of “very good” or “good” adherence from the caregiver, and having latest VL < 1000 copies/ml. This conservative VL cutoff was selected to encompass only true treatment failures and exclude possible transient VL blips. Because of the different laboratory sensitivities at the local hospitals, we chose the highest VL threshold for homogeneity purpose. Participants with missing measures of adherence were excluded from the analysis.

ART adherence was considered as a binary variable (adherence versus suboptimal adherence). The concordance between the different measures of adherence was evaluated using the kappa test. Analysis with logistic regression models was performed using suboptimal adherence as the outcome. A complete list of independent variables included in the analysis is presented in S1 Table. First, each independent variable was assessed individually for its univariate relationship with the outcome. Variables with moderate to strong association (p<0.10) with suboptimal adherence were considered in the multivariate analysis. Variables in this subset were sequentially added in a stepwise forward fashion based on Wald score to build the multivariate model. At each step, the newly added predictor remained in the model if its regression coefficient was significant (p<0.05), and would be removed from the model if p≥0.10. Adolescent age (categorical), gender and years on ART (categorical) were entered as the first step in all multivariate analyses. When there was strong correlation between two or more predictors, we selected only one predictor to include in the analysis based on Wald scores in univariate analysis. Of note, lines of ART treatment and adolescent obedience rated by caregivers were not included in the multivariate analysis despite demonstrating strong associations in the univariate analysis, as these variables were considered to be likely consequences rather than causes of suboptimal adherence. Statistical analyses were conducted using the Statistical Package for the Social Science (SPSS) version 20.0. Statistical significance was set at 0.05. P-values and Wald statistic were reported for all relevant regression coefficients.

### Qualitative component

12 in-depth interviews (IDIs) were conducted in 2014 among families who had participated in the TEEWA study in Chiang Mai, Thailand, including 6 perinatally infected adolescents/youth (aged 18–23) and 6 caregivers of perinatally infected adolescents/youth (aged 12–21). The informants were recommended and contacted by local Community Advisory Board based on representative characteristics of adolescent age, gender and caregiver type. All adolescent informants were aged ≥18 years and fully aware of their HIV status. While the informants were from the same participant pool of TEEWA survey, analysis of the IDIs was not linked to their quantitative counterparts given the anonymised data in the survey.

All interviews were conducted by the first author (female) with the third author (female) or another PHPT staff (male) as the Thai interpreter. The first author was a medical student with previous experience of ethnographic work, however no relationship was established between the first author and the participants prior to the interviews. Both the third author and the other interpreter worked for the TEEWA study as research nurses and knew the adolescent/youth participants; they were both Thai natives and therefore served as cultural brokers at the same time. The interviews took place in private rooms at PHPT office or at a local community health center. Each interview lasted about 90 minutes. An interview topic guide was developed based on review of relevant literature and experiences from PHPT staff who worked closely with the perinatally infected adolescent population. In addition, the informants were also encouraged to discuss freely the issues they believed as most important to ART adherence. All interviews were audio-recorded. The first author worked closely with a Thai interpreter who did not participate in the interviews to transcribe and translate all interview contents verbatim into English. We did not obtain participants’ comments or corrections on the transcripts.

The interviews were analyzed using the thematic content approach. The first author and a research assistant went through an iterative process involving coding the transcripts separately first and reconvening to reach consensus, until all themes and subthemes identified were deemed coherent by both parties and the principal investigators of the study. MAXQDA 11 was used to facilitate coding.

## Results

### Quantitative findings

**Fig 1:**
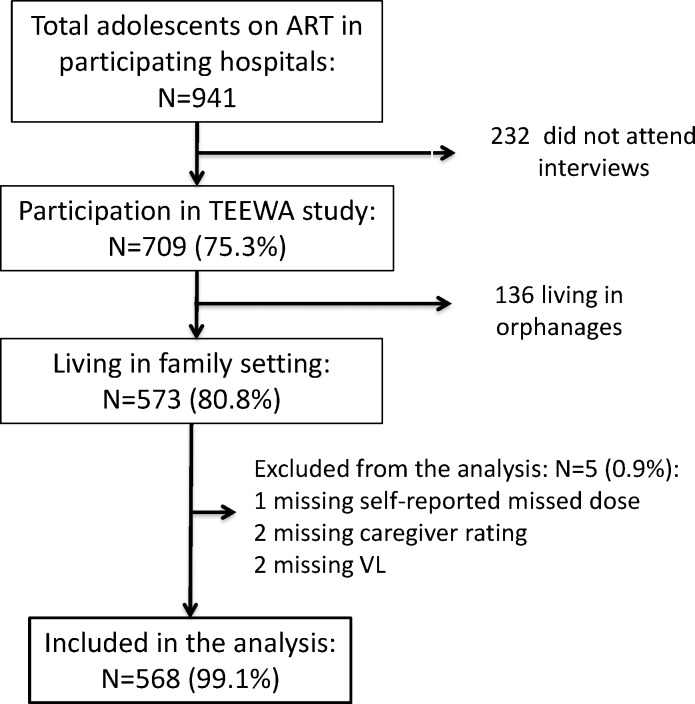
Data included for analysis from the Teens Living With Antiretrovirals (TEEWA) database.

A total of 941 perinatally HIV infected adolescents (aged 12 to 19) were receiving ART from the 20 participating hospitals in the TEEWA study. 709 (75.3%) pairs of adolescents and their caregivers participated in the survey. 136 adolescents living in orphanages were excluded from this analysis because they generally receive ART under direct observation, as indicated by the high rate (97%) with a VL <1000 copies. Five additional adolescents were excluded because adherence information was missing (1 missing self-reported missed dose, 2 missing caregiver rating, and 2 missing VL). Therefore, a total of 568 participants living in family setting were considered in the analysis (Fig 1). The adolescents who did not participate in the survey were slightly older than the participants (median age 15.7 vs. 14.4 years) but there was no significant difference in terms of gender. Characteristics of participants included in the analysis are presented in Table 1. The median age at time of survey (2010–2012) was 14.4 years (Interquartile Range (IQR), 13.1–16.0), 42% were male, and 59% were receiving care in northern Thailand. Only 31.5% of the adolescents were living with biological parents, and 86.3% had lost one or both parents. The median age at HIV diagnosis and ART initiation was 8.0 years (IQR, 5.0–10.0) and 9.0 (IQR, 7.0–11.2) years, respectively. Predominant treatment modality was non-nucleotide-reverse transcriptase inhibitor (NNRTI)-based first-line ART (73.9%) and twice daily ART intakes (94.7%).

**Table 1:** Characteristics of TEEWA study participants included in quantitative analysis.

Characteristics (N = 568)		Median (Interquartile Range) or N (%)		
		Adherent participants (n = 293)	Participants with suboptimal adherence (n = 275)	All participants (n = 568)
Age (years)		14.4 (13.1–16.0)	14.3 (13.1–16.0)	14.4 (13.1–16.0)
Male		124 (42.3)	113 (41.1)	237 (41.7)
Puberty^a^		198 (68.0)	179 (65.1)	377 (66.6)
Geographic region	North	173 (59.0)	159 (57.8)	332 (58.5)
	Northeast	30 (10.2)	35 (12.7)	65 (11.4)
	Center and South	90 (30.7)	81 (29.5)	171 (30.1)
Caregiver type	Biological parents	110 (37.5)	69 (25.1)	179 (31.5)
	Grandparents	108 (36.9)	105 (38.2)	213 (37.5)
	Other family members	75 (25.6)	101 (36.7)	176 (31.0)
Orphan status	Both parents alive	46 (15.7)	32 (11.6)	78 (13.7)
	Mother died or unknown	59 (20.1)	48 (17.5)	107 (18.8)
	Father died or unknown	64 (21.8)	48 (17.5)	112 (19.7)
	Both died or unknown	124 (42.3)	147 (53.5)	271 (47.7)
Caregiver education	None	47 (16.0)	36 (13.1)	83 (14.6)
	Primary school	191 (65.2)	187 (68.0)	378 (66.5)
	Secondary school and above	55 (18.8)	52 (18.9)	107 (18.8)
Suboptimal adherence (nonadherence by one or more of the three measures)		-	-	275 (48.4)
	Self-reported missing dose in past 7 days	-	-	197 (34.7)
	Suboptimal adherence rated by caregiver	-	-	43 (7.6)
	Most recent viral load ≥ 1000 copies/ml	-	-	101 (17.8)
Age at HIV diagnosis (years)		8.0 (6.0–11.0)	8.0 (5.0–10.0)	8.0 (5.0–10.0)
Age at ART initiation (years)		9.2 (7.1–11.0)	8.9 (6.8–11.5)	9.0 (7.0–11.2)
Years on ART		5.7 (4.0–7.0)	6.0 (4.1–7.3)	5.9 (4.0–7.1)
Type of ART	Non nucleotide reverse transcriptase inhibitor (NNRTI)-based	242 (82.6)	178 (64.7)	420 (73.9)
	Protease Inhibitor (PI)-based	41 (14.0)	76 (27.6)	117 (20.6)
	Dual PI	8 (2.7)	16 (5.8)	24 (4.2)
	Treatment interruption or only dual/mono therapy	2 (0.7)	5 (1.8)	7 (1.2)
Most recent absolute CD4		701 (523–910)	558 (350–737)	635 (441–859)
Most recent CD4%^b^		27 (22–32)	23 (16–23)	25 (19–30)
Most recent VL		40 (40–50)	50 (40–7425)	40 (40–80)
Time from most recent CD 4 to interview (months)^c^		5 (3–8)	5 (3–7)	5 (3–8)
Time from most recent VL to interview(months)^d^		8 (4–12)	6 (3–11)	7 (4–12)
Presence of side effects		35 (11.9)	39 (14.2)	74 (13.0)
Frequency of daily intake^e^	1	4 (1.5)	8 (3.1)	12 (2.3)
	2	257 (95.5)	241 (93.8)	498 (94.7)
	≥ 3	8 (3.0)	8 (3.1)	16 (3.0)
Number of daily tablets^f^		2.0 (2.0–6.0) 4.1 (mean)	4.0 (2.0–7.0) 4.5 (mean)	3.0 (2.0–7.0) 4.3 (mean)

a. 2 missing values

b. 1 missing value

c. 1 missing value

d. 1 missing value

e. 42 missing values

f. 56 missing values.

Median (Interquartile Range) is presented for continuous variables, N (%) for categorical variables.

In terms of ART adherence, 197 (34.7%) adolescents reported missed doses in the past 7 days; 43 (7.6%) caregivers gave suboptimal rating of the adolescent’s adherence (“average”, “poor”, or “very poor”); and 101 (17.8%) adolescents had a VL ≥ 1000 copies/ml (Table 1). A total of 275 (48.4%) adolescents had evidence of nonadherence by one or more of the three measures, and were therefore considered to have suboptimal adherence in the analysis. Agreement between the three adherence measures is shown in Table 2, among which the highest observed agreement was between the VL measures and caregivers’ rating of adherence (at 80.6%, kappa = 0.145). Adolescents’ self-reported missed doses in the last 7 days agrees poorly with VL measures (61.9%, kappa = 0.052) and caregiver ratings (64.4%, kappa = 0.039).

**Table 2:** Agreement between three different ART adherence measures.

	Caregiver rating		Kappa	% concordance
	Very good or good	Suboptimal		
VL<1000	441	26	0.145	80.6
VL≥1000	84	17		
	Adolescent self-reports		Kappa	% concordance
	No missed dose in the last 7 days	Missed dose in the last 7 days		
VL<1000	311	156	0.052	61.9
VL≥1000	60	41		
	Caregiver rating		Kappa	% concordance
	Very good or good	Suboptimal		
No self-reported missed dose in the last 7 days	347	24	0.039	64.4
Self-reported missed dose in the last seven days	178	19		

Table 3 presents results from univariate and multivariate analysis of factors associated with suboptimal adherence. Seven variables were found independently associated with suboptimal adherence in the multivariate analysis: younger age, having grandparents or extended family members as the primary caregiver, poor intellectual ability as assessed by the caregiver, adolescent self-reported having a boy/girlfriend, unhappiness, frequent online chatting, and easiness in asking doctors questions. Other predictors, such as frequent arguments between adolescent and caregiver, discrimination from friends, working and alcohol drinking were associated (p<0.05) with suboptimal adherence in the univariate analysis, but the association did not remain significant in the multivariate model (S1 Table). Of note, caregiver education level, household financial status, ART treatment duration, disclosure of HIV status to the adolescent, or the presence of ART side effects were not associated with suboptimal adherence in the univariate analysis (Table 3 and S1 Table). Given that the great majority of caregivers rated their adolescent as adherent (92.4%) by the current definition, we also performed an analysis using a more stringent definition of adherence (no self-reported missed dose in the past 7 days, receiving a caregiver rating of adherence as “very good” only, and having latest VL < 1000 copies/ml). The results were consistent with the present analysis.

**Table 3:** Odds ratio of selected suboptimal adherence predictors in univariate and multivariate analysis.

Predictor (N)	Category	Suboptimal adherence n/N (%)	Univariate analysis		Multivariate analysis	
			OR^a^ (95% CI)	*P*	aOR^b^ (95% CI)	*P*
Adolescent gender (568)	Male	113/237 (47.7)	1		1	
	Female	162/331 (48.9)	1.052 (0.753–1.469)	0.766	1.057 (0.710–1.574)	0.784
Adolescent age (568)	12–13	123/249 (49.4)	1		1	
	14–15	83/177 (46.9)	0.905 (0.615–1.330)	0.610	0.648 (0.404–1.040)	0.072
	16–19	69/142 (48.6)	0.968 (0.641–1.462)	0.878	0.575 (0.341–0.968)	0.037
Years on ART (568)	0–4	104/216 (48.1)	1		1	
	5–6	86/188 (45.7)	0.908 (0.614–1.344)	0.629	1.062 (0.670–1.684)	0.797
	≥7	85/164 (51.8)	1.159 (0.772–1.739)	0.477	1.011 (0.627–1.631)	0.963
Adolescent’s intellectual ability assessed by caregiver (547)	Good, very good	109/259 (42.1)	1		1	
	Average	116/228 (50.9)	1.425 (0.996–2.039)	0.052	1.368 (0.909–2.060)	0.133
	Poor, very poor	38/60 (63.3)	2.377 (1.331–4.246)	0.003	2.886 (1.410–5.905)	0.004
Caregiver type (568)	Parents	69/179 (38.5)	1		1	
	Grandparents	105/213 (49.3)	1.550 (1.035–2.320)	0.033	1.649 (1.027–2.650)	0.039
	Other family members	101/176 (57.4)	2.147 (1.405–3.281)	<0.001	2.100 (1.277–3.456)	0.003
Easiness to ask doctors questions reported by adolescent (567)	Very easy	76/134 (56.7)	1		1	
	Easy	94/204 (46.1)	0.652 (0.421–1.011)	0.056	0.510 (0.302–0.861)	0.012
	Ok	92/210 (43.8)	0.595 (0.384–0.921)	0.020	0.400 (0.231–0.690)	0.001
	Very difficult, difficult	12/19 (63.2)	1.308 (0.485–3.531)	0.596	0.625 (0.184–2.125)	0.452
Happiness reported by adolescent (568)	Very happy	34/98 (34.7)	1		1	
	Happy	94/189 (49.7)	1.863 (1.125–3.084)	0.016	2.420 (1.331–4.398)	0.004
	Average	84/174 (48.3)	1.757 (1.054–2.929)	0.031	2.716 (1.448–5.097)	0.002
	Unhappy or very unhappy	63/107 (58.9)	2.695 (1.529–4.751)	0.001	3.849 (1.896–7.815)	<0.001
Has a boy/girlfriend reported by adolescent (567)	No	199/441 (45.1)	1		1	
	Yes	76/126 (60.3)	1.848 (1.235–2.766)	0.003	1.965 (1.199–3.220)	0.007
Chat on internet (567)	No	127/287 (44.3)	1		1	
	Sometimes	129/237 (54.4)	1.505 (1.065–2.127)	0.021	1.842 (1.201–2.824)	0.005
	Often	19/43 (44.2)	0.997 (0.523–1.902)	0.994	1.495 (0.687–3.252)	0.311
Caregiver education (568)	Never	36/83 (43.4)	1		—	—
	Primary	187/378 (49.5)	1.278 (0.792–2.063)	0.315	—	—
	Secondary and above	52/107 (48.6)	1.234 (0.694–2.197)	0.474	—	—
Financial situation (568)	Ok, good, very good	179/360 (49.7)	1		—	—
	Very difficult, difficult	96/208 (46.2)	0.867 (0.616–1.220)	0.412	—	—
Arguments with caregiver reported by adolescent (567)	Never or rarely	119/267 (44.6)	1		—	—
	Sometimes	103/213 (48.4)	1.165(0.812–1.671)	0.408	—	—
	Often or very often	52/87 (59.8)	1.848 (1.130–3.022)	0.014	—	—
Discrimination from friends reported by caregiver (568)	No or unclear	203/442 (45.9)	1		—	—
	Yes	72/126 (57.1)	1.570 (1.053–2.340)	0.027	—	—
Disclosure of HIV status reported by caregiver (568)	Adolescent does not know	30/66 (45.5)	1		—	—
	Formal disclosure	170/347 (49.0)	1.153 (0.680–1.955)	0.598	—	—
	Adolescent knows without formal disclosure	75/155 (48.4)	1.125 (0.631–2.005)	0.690	—	—

^a^ OR = Odds Ratio

^b^ Adjusted for adolescent age (categorical), gender and years on ART (categorical)

### Qualitative findings

Table 4 provides the characteristics of in-depth interview participants in the qualitative study. Table 5 lists the themes and subthemes associated with ART adherence identified through analysis of the IDIs. Some important findings and quotes are highlighted below.

**Table 4:** Characteristics of in-depth interview participants in qualitative study.

Interview Participants	Adolescent/Youth Age (years)	Adolescent/Youth Gender	Type of Primary Caregiver
Adolescent /youth informants	21	male	Biological mother (HIV-infected)
	23	female	
	18	male	Grandparents and relatives (parents deceased)
	21	female	
	22	male	Adopted mother (parents deceased)
	20	female	Orphanage staff (parents deceased)
Caregiver informants	13	female	Biological mother (HIV-infected)
	21	male	
	18	female	Biological father (HIV-uninfected)
	12	male	Grandparents (parents deceased)
	17	male	
	0–18	male & female	Faith-based orphanage manager

**Table 5:** Themes associated with ART adherence from analysis of in-depth interviews.

Knowledge and perception	-Understanding of HIV/AIDS disease, how ARTs work and the consequence of nonadherence -Perceived benefits and burdens of treatment
Childhood experience	-Presence or absence of biological parents -Home stability and financial concerns -Adverse experience with severe illness -Disclosure of HIV status
Interpersonal relationships	-Communication, counseling and/or conflicts with caregivers -HIV-infected peers vs. non-infected peers -Boy/girlfriends -Others: support from doctors, teachers, role models, non-profit groups, religious groups
Daily environment	-Busy schedules and activity engagement -School environment and lack of privacy -Experience of discrimination and/or internalized stigma of HIV/AIDS
Self-help skills	-Independence in taking ARTs, with use of different modalities of reminders (alarm clock, pill box, etc.) -Incorporate medication taking into daily schedule -Participate in treatment management with health providers -Strategies in disclosing HIV status to others, including in romantic relationships -Willingness to seek help and/or psychosocial counseling when needed

### Knowledge and perception

All informants demonstrated good knowledge regarding HIV/AIDS and ART, including the consequences of developing resistance with suboptimal adherence. They reported multiple sources of training, including health care providers, support groups, and community health educators. The adolescents reported improvement in skin conditions (absence of rashes), strength and overall health as benefits of treatment. They also expressed concerns about side effects that altered body shape (lipodystrophy) or interfered with normal activities (headaches, gastrointestinal disturbances). All adolescent informants used the phrase “bua ya” (“feeling bored”) when describing their experience with long-term treatment, along with the feeling that “the disease is not curable”.

Adolescent 1: The side effects (headache and nausea) happen an hour after taking the medicine. I have to rest. Because of this I’m not taking the medicine on time. I cannot do my activities as normal.

### Childhood experience

Events reflecting household instabilities are frequently reported, including HIV-related severe illness or death of biological parents, frequent changes of primary caregivers, substance abuse and violence within the household, and financial difficulties. Additionally, uncoordinated disclosure of HIV status during childhood was a major source of confusion and negative feelings toward the medication.

Adolescent 3: (When I learned about my infection), I wanted to throw the medicine over the fence. I thought that I was not a normal person. I felt like I was a failure. (I wondered) why I didn’t have parents, and why I had to be like this.

### Relationship with caregivers

Tensed relationships or lack of communication with the caregiver were cited by more than half of the informants as potential reasons for suboptimal adherence, especially when the caregiver was a distant family member. Opposing attitudes leading to poor adherence may result from excessive reminders of taking ART from the caregivers. Additionally, caregivers were often reluctant to address sensitive topics such as HIV-related discrimination and adolescent’s relationship with peers or with boy/girlfriends.

Adolescent 5: (My aunt and grandfather) asked me, “Did you take your medicine?” I answered, “Yes, I did.” But actually I didn’t. They remind me many times, but I did not follow. We don’t talk much to each other. And I think I don’t have things to talk to them.

Caregiver 2: I did not take (others’ discrimination towards my grandson) seriously…(I said to my grandson), “If you don’t want to play with other children, you can stay with me… Saturday, Sunday, stay with me and watch TV.” He did not go out anywhere, just stay in the house during the weekend.

### Psychosocial support

The informants identified peer groups, positive adult role models, good relationship with doctors and resources from non-profit organizations as important factors for adherence. Among these, peer group was well received by almost all informants as a major source of psychosocial support. The peer group activities were frequently described by the adolescents as fun, relaxing, and mind-opening. Becoming a peer group leader also helped them find values and self-esteem in extending their responsibilities to others in the society.

Adolescent 2: [Being in a peer group] makes me feel that I have to take care of myself. It cheers me up and I realized that I should not waste time with naughty friends. I can do the activity that can help younger people and cheer them up.

### Forgetfulness, busy schedule and delayed medication

Engagement in extra-curriculum activities or busy school schedules were the most commonly cited reasons by both adolescents and caregivers for forgetting ART.

Adolescent 4: (I forgot medicine) at the time I was very busy. I like to read and write the novel. It was the time when I enjoy (myself), and I forget (the medicine).

Adolescent 6: Sometimes I don’t know how long I need to stay (at school), and I forget (to bring) the medicine.

### Discrimination and the need for privacy

Almost all informants reported direct or indirect encounters of discrimination or mistreatment due to HIV infection. The adolescents found it particularly difficult to disclose their HIV status to uninfected peers because of the associated stigma, especially when in a romantic relationship. 5 out of 6 adolescent informants reported having to hide to take the medicine in fear of revealing their HIV status, and expressed concerns about lack of privacy when taking medications at school or with friends. These concerns were confirmed by the caregiver informants as well.

Adolescent 2: They (other adolescents) fear that people would notice when they are taking medicine, and ask what kind of medicine they are taking. The teacher recommended the technique (to say) that it is the medicine for health, for beauty. And some are afraid that their boy/girlfriend would discover the truth. So they decide to stop the medicine, as they feel they are still healthy.

### Self-help skills

The interviews revealed various levels of skills among the adolescents. While some are actively engaged in their treatment management, others feel reluctant to ask for help, stating that they did not know what to say or who to talk to at times of distress. Informants discussed important skills related to ART adherence, including the ability to assume individual responsibility of ART taking, to disclose HIV status to others and handle situations of discrimination, and to seek help in a timely manner when needed.

Adolescent 1: I switched (my regimen) from twice daily to one time a day last year. I asked the doctor to change. Because I saw my friend take the ARV once a day, I think it would be more convenient for me. That’s why I asked the doctor to change.

Adolescent 5: I didn’t talk to anyone (about feeling sad). I didn’t know how to speak it out.

## Discussion

Our study is among the first ones that used both quantitative and qualitative approaches to identify factors influencing ART adherence among the unique group of perinatally infected adolescents in Thailand. We excluded adolescents infected behaviorally and adolescents living in institutions to achieve a more homogeneous study population, as these two groups had very different experiences of the HIV/AIDS disease and supervision of ART taking. Both our quantitative and qualitative analyses consistently showed findings emphasizing the psychosocial aspects, especially the adolescents’ interpersonal relationships and adaptive abilities.

We used a stringent definition of adherence, combining all three measures including the objective VL level, adolescents’ self-reports and caregivers’ evaluation of overall adherence. We recognized poor agreements between adolescents’ self-reported adherence and VL measures or caregiver evaluations (Table 2), which suggest a potential time gap between the onset of skipped doses and the presentation of virological failure, and that the caregivers might be often unaware of these skipped doses. Similar discordance of adherence reports was observed in other studies, with increasing discrepancies between the child and the caregiver by late adolescence[26]. In a recent cohort study analyzing models of latent trajectories of average ART adherence, immunological failure and mortality both increased with increased variance of adherence, emphasizing the importance of consistent ART intake[9]. Therefore, our stringent definition allowed us to identify not only nonadherence but also suboptimal adherence that may likely jeopardize response to ART treatment in this vulnerable population in the long term.

Our approach of including both quantitative and qualitative components provided a comprehensive evaluation of the interpersonal dynamics that might influence ART adherence. The quantitative results were supported by qualitative findings to reveal the underlying mechanisms by which the identified factors influenced adherence. In the multivariate analysis, the more distant the relationship with the caregiver, the higher the odds of suboptimal adherence, which increased by 65% when the caregivers were grandparents (adjusted Odds Ratio (aOR) = 1.649, p = 0.039) and more than doubled with extended family members (aOR = 2.100, p = 0.003) as compared to living with biological parent(s). This is supported by the qualitative findings of tense relationships and lack of communication between adolescents and more distant family members, indicating that undesired household situations contributed to poorer adherence. Similarly, the fear in disclosing HIV status in a romantic relationship not only psychologically influenced the adolescents’ self-esteem, but the need to conceal one’s HIV status also added significant behavioral barriers to timely medication taking and help seeking, which may explain the increased rate of suboptimal adherence (aOR = 1.965, p = 0.007) associated with having a boy/girlfriend. The negative effect on adherence from frequent chatting on the Internet (aOR = 1.842, p = 0.005) correlates well with the qualitative findings that adolescents often missed or delayed a dose when immersed in other activities. These are representative challenges encountered by the adolescents in daily life, as they not only face an external world with complicated interpersonal relationships and exciting (although at times risky) activities to explore, they also need to come to terms with the internal self who desire to be equal and normal despite growing up with HIV. The significance of their cognitive and adaptive abilities could not be overlooked in this unique period of development. Arguably, poor intellectual ability (aOR = 2.886, p = 0.004) likely impairs adolescent’s adaptation in integrating ART taking into an already challenging daily life, whereas these challenges may be alleviated by increased maturity with age (aOR = 0.575, p = 0.037). It is also not surprising that happiness ("Mi Kuam Suk", or to have happiness in Thai), which can be seen as a surrogate for overall satisfaction in life and a healthy mental status with strong motivation, turned out to be one of the strongest factors associated with adherence, with the rate of suboptimal adherence almost quadrupled when an adolescent reported to be unhappy or very unhappy (aOR = 3.849, p<0.001). Interestingly, adolescents who found it less easy to ask their doctors questions were shown to have better adherence (aOR = 0.400, p = 0.001). This suggests that those adolescents who felt more at ease with their doctors were more comfortable in not being able to strictly follow treatment instructions or to truthfully report missed doses. This observation is consistent with a cognitive dissonance hypothesis[27] (not tested in this study) that the existence of psychological discord with the doctor may lead to greater motivation in the adolescent in trying to reduce uneasiness by achieving consonance through better adherence. The cognitive dissonance mechanism may also help explain those adolescents’ greater degree of tolerance of discomfort in boy/girlfriend relationships through concealment or untruthfulness about their HIV status and suboptimal ART adherence.

Our findings are consistent with a number of studies published in Thailand and worldwide, which highlighted the importance of psychosocial factors in ART adherence[28–31]. The presence of a biological parent is one of the most frequently reported factors associated with good adherence[19, 21, 32–34]. Stigma and lack of privacy have also been reported across different cultures to significantly impair ART adherence[19, 32, 35–38]. Similarly with a recent systematic review[39], we did not find a clear association between ART adherence and social economic status, as indicated by the caregiver’s financial situation or education, suggesting that socioeconomic disadvantages may not necessarily be the absolute barriers to adherence, if an effective support system is in place.

Our study also revealed several factors unique to the perinatally infected adolescents. While substance abuse was more frequently a barrier to adherence among behaviorally infected youth[18, 40, 41], exposure to alcohol, drug use, and other risk-taking behaviors were not significantly associated with ART adherence in our multivariate analysis, suggesting different targets for future intervention between these two groups. Studies on disclosure of HIV status to perinatally infected adolescents have so far yielded conflicting results regarding influence on ART adherence[42–45]. While our quantitative analysis did not demonstrate an association between disclosure and adherence, our data on disclosure was collected from caregivers alone, which might not be accurate to ascertain unplanned versus formal disclosure, as our IDIs suggested that caregivers might be unaware that adolescents had already discovered their HIV status by themselves.

There are a number of limitations to our study. We did not perform a random selection of hospitals based on regional HIV prevalence, which affected our ability to generalize the findings at the country level. However, considering that about 9,000 HIV-infected adolescents aged 12–19 years were receiving ARTs in Thailand in 2012 per National Health Security Office (NHSO), the 941 adolescents followed in our 20 participating hospitals represented roughly 10% of this population. The hospitals spanned a wide range of geographic locations as shown in Table 1 as well as different care levels from community hospitals to tertiary centers to reach both urban and rural populations, allowing a good representation of adolescents born with HIV, living in family setting and receiving ARTs in Thailand. To prevent unintended HIV disclosure in the TEEWA study, the detailed practice of medication taking and HIV status disclosure could not be directly explored from the adolescents. Since all adolescents remained in their pediatric care phase, our results may not reflect the situation after transition into adult care, which may negatively affect adherence. Finally, as a cross-sectional survey, causal relationships between associated factors and suboptimal adherence cannot be definitively established, and further studies are required. The qualitative component of the study was limited by a small sample size and potential selection biases, therefore cautions should be taken in generalizing the findings. Given a time lapse of 2–4 years between TEEWA survey and the IDIs, adolescents’ insights during the interviews might have changed or evolved as compared to their perspectives during the time of the survey. The aim of the IDIs, however, was not to draw definitive conclusions, but rather to provide a qualitative dimension in our understanding and to facilitate the interpretation of the quantitative data.

As elegantly pointed out by other qualitative studies[31, 38, 46], we must understand factors influencing ART adherence by situating adolescents in the complex social and interpersonal contexts they face on a daily basis. Our IDIs revealed that skipping or stopping ARTs were often conscious decisions made by the adolescents when conflicts arise between treatment taking and other social concerns. Guidance and support from caregivers, peers and health providers likely play a crucial role in helping the adolescents develop necessary skills to manage complicated social circumstances as they grow up with HIV and long-term medication taking. Suggestions from our findings are two-fold: at the individual level, future interventions should emphasize enhancing adolescents’ mental health as well as building adaptive skills in recognizing and addressing challenging situations related to ART taking. In particular, peer groups can provide an understanding and judgment-free environment for emotional support, trouble-shooting and skill building, as evidenced by other studies[29, 47] as well as the vehement support from all our IDI participants. At the interpersonal level, integrated HIV management should also target caregivers and health providers to facilitate timely recognition of age-specific challenges on ART adherence and develop strategies in providing constructive counseling as they accompany the children through the tumultuous period of adolescence.

## Conclusion

Perinatally infected adolescents face unique challenges in ART adherence as they come of age. Using both quantitative and qualitative approaches, our findings highlighted the importance of both individual and interpersonal psychosocial factors in ART adherence, especially in terms of the adolescents’ expressed level of happiness, their relational distance from key caregivers, and their cognitive and adaptive abilities to tolerate discomfort in their relationships with their boy/girlfriends or medical doctors. The knowledge of factors influencing ART adherence as noted in this study could benefit clinicians in providing better care for those adolescents with highest risk for suboptimal adherence. As current ART regimen requires lifelong medication for viral suppression, more proximal and unfailing caregiving relationships are crucial for age-appropriate psychological, emotional, and behavioral supports across the lifespan. We recommend that future interventions be better targeted at adolescents’ cognitive and adaptive coping skills. These interventions need to recognize the adolescents’ hopes, desires, as well fears and challenges related to HIV, in fitting their responsibilities with regards ART adherence in their daily lives. This involves promotion of better targeted individual and group counseling programs that emphasize improved communication and interpersonal relational supports with peers, caregivers, and health providers. Such an evidence-based approach as informed by this study will likely not only enhance ART adherence but improve the quality of their lives and aspirations.

## Supporting information

S1 File TEEWA Study Adolescent Questionnaire (English). (PDF)

S2 File TEEWA Study Adolescent Questionnaire (Thai). (PDF)

S3 File TEEWA Study Caregiver Questionnaire (English). (PDF)

S4 File TEEWA Study Caregiver Questionnaire (Thai). (PDF)

S5 File TEEWA Study Questionnaire Life Event Table (English). (PDF)

S1 Table Odds ratio and Wald score of suboptimal adherence predictors in univariate analysis. (DOCX)
