# Supplementary material for: Factors influencing antiretroviral treatment suboptimal adherence among perinatally HIV-infected adolescents in Thailand
Source: PLoS One. 2017 Feb 16;12(2):e0172392. doi: 10.1371/journal.pone.0172392 (PMC5312953; doi:10.1371/journal.pone.0172392)
Supplement: S1 File — (PDF) [file pone.0172392.s001.pdf]

Institut National d'Etudes Démographiques, Paris
Faculty of Associated Medical Sciences, Chiang Mai University, Chiang Mai

TEENAGERS QUESTIONNAIRE

Girls

Self-administered

ID Number: [ ]

Date: |__|__| day |__|__| month |_2_|_5_|_5_|_ _| yr

Please introduce the study and ask for ascent

Self administred : ☐ With help: ☐

Site: .... Code : |__|__|

YOU ARE LIVING IN AN ORPHANAGE

How old are you? |___|___|years old today

What is your date of birth (year that you were born) |___|___| |___|___| |___|___|___|___|

Day Month Year

How many brothers and sisters do you have? |___|sisters |___| brothers

How many children are sharing the same bedroom including yourself? |___|___|

Among them, are there children you feel close to

- ☐ None
- ☐ One or two, only
- ☐ Three or four
- ☐ Many
- ☐ Most of them

How are the relations you have with the adult(s) in charge of you at the orphanage?

- ☐ Very good
- ☐ Good
- ☐ OK
- ☐ Difficult
- ☐ Very difficult

NOW GO TO PAGE 4

YOU ARE LIVING IN A FAMILY/PRIVATE HOME

How old are you? |___|___|years old today

What is your date of birth (year that you were born) |___|___| |___|___| |___|___|___|___|

Day Month Year

At home, I live with (Relationship)

Nobody ☐ **Go to Page 4**

	I share my bedroom with her/him?	I feel close to her/him?
1.	☐ Yes	☐ Yes
2.	☐ Yes	☐ Yes
3.	☐ Yes	☐ Yes
4.	☐ Yes	☐ Yes
5.	☐ Yes	☐ Yes
6.	☐ Yes	☐ Yes
7.	☐ Yes	☐ Yes
8.	☐ Yes	☐ Yes

At home, who is looking after you the most? ....

Do you help with the household chores (*cleaning, shopping, cooking, etc.*)?

- ☐ Yes often
- ☐ Yes sometimes
- ☐ Rarely
- ☐ Not at all

Is there a particular task in the household that you are personally responsible for? *(watering the garden, washing, etc...)?*

- ☐ No
- ☐ Yes, what?..................

Do you regularly help the adults with their work (*in a shop, farm, etc.*)

- ☐ No
- ☐ Yes, what?...............

Is there somebody in the household that you regularly look after

- ☐ No
- ☐ Yes, who?................

SCHOOL

Are you currently attending school?
- ☐ Yes, what grade are you in? ……
- ☐ No, in which grade did you stop ……..

Do (or did) you like school?
- ☐ Very much
- ☐ A little
- ☐ It is Ok
- ☐ I don't like it
- ☐ I don't like it at all

Do (or did) you skip (leave) school because you were ill?
- ☐ Yes regularly
- ☐ Yes for a long time
- ☐ Sometimes
- ☐ Rarely

What kind of student are (or were) you?
- ☐ Very good
- ☐ Good
- ☐ Fair
- ☐ Bad
- ☐ Very bad

Do (or did) you have friends at school?
- ☐ A lot
- ☐ Some
- ☐ 2 or 3
- ☐ None

WORK

Do you regularly work for money?

☐ No ☐ Yes

What do you do ? ……………………………….

How many days a week do you work? |__|

How much do you earn? |__|__|__|__| per day
or
|__|__|__|__| per month

Do you give money to your household
☐ No
☐ Sometimes
☐ Regularly

Do you like your work ? ☐ No ☐ Yes

Do you get tired at work? ☐ No ☐ Yes

Do you have friends at work?
☐ Many
☐ Some
☐ Few
☐ None

FUTURE

Do you plan to go to college, vocational college, university?
☐ No
☐ Yes
☐ Don't know

What subject would you like to study? …………………………… ☐ Don't know

What kind of occupation would you like to have, ideally? …………………………….☐ Don't know

Do you think that you will get married or have a partner in the future?
☐ No
☐ Yes ☐Don't know

Do you think that you will get children in the future?
☐ No
☐ Yes ☐ Don't know

Do you think that you will live outside your hometown in the future?
☐ No
☐ Yes ☐ Don't know

HEALTH

How is your health at the moment?

- ☐ Very Good
- ☐ Good
- ☐ Fair
- ☐ Poor
- ☐ Very Poor

Compared to your friends, are you :

**Height**	**Weight**
☐ Taller	☐ Chubbier
☐ Average	☐ Average
☐ Smaller	☐ Skinnier

Have you been hospitalized ☐ No ☐ Yes

The last time, how old were you? |__|__|

☐ Don't remember

How long did you stay in hospital?

|__|__|months or |__|__|Days

Do you take regularly any treatment?

- ☐ No → **go to page 8**
- ☐ Yes

How old were you when you started treatment? |___|___|years old ☐ Don't know

Do you have side effects from your treatment?

- ☐ No
- ☐ Yes, specify:..............................

Is somebody giving you your treatment?

- ☐ No, I take it by myself
- ☐ Yes

Who ? ....

Is somebody reminding you to take your treatment?

- ☐ Never
- ☐ Sometimes
- ☐ Always

Who ? ....

Do you find it easy or difficult to remember to take your treatment?	☐ Very Easy<br>☐ Easy<br>☐ OK<br>☐ Difficult<br>☐ Very difficult
Do you use any tricks to prepare or remember to take your treatment on time? (tick all that apply)	☐ None<br>☐ Clock/watch alarm<br>☐ Cell phone alarm<br>☐ National anthem<br>☐ TV / radio program<br>☐ Use pill box w/ compartments<br>☐ Other: ....
During the last week (7 days), did you ever forget to take your treatment?	☐ Never<br>☐ I forgot one dose<br>☐ I forgot several doses<br>☐ Don’t know
If you ever forget to take your medication, what was the main reason?	☐ I was sick<br>☐ I was playing<br>☐ I was too busy<br>☐ I was fed up<br>☐ I was not at home<br>☐ It was a festival/holidays<br>☐ I was not given them<br>☐ There wasn’t enough privacy<br>☐ Other reasons, specify: ....<br>...<br>.........
Do you get scolded when you forget to take your treatment (*nurse, doctor, family*)?	☐ Yes<br>☐ No

REPRODUCTIVE HEALTH

Have you already begun menstruation?
- ☐ No
- ☐ Yes, it started at |__|__| years old

Have you received any sexual education/family planning?
- ☐ No
- ☐ Yes

Where:
- ☐ at school
- ☐ in a children's camp
- ☐ at the Hospital
- ☐ At home
- ☐ Others, specify: ....

Have you ever had sexual intercourse?
- ☐ Don't want to answer **go to page 9**
- ☐ No → **go to page 9**
- ☐ Yes, the first time at |__|__| years

With whom was it the first time?
- ☐ Spouse
- ☐ Female CSW
- ☐ Boy/girl friend
- ☐ Male CSW
- ☐ Occasional partner
- ☐ Other (specify): ....
....

Age of your partner at first sex |__|__| years ☐ Don't know

Did you use a condom at this time?
- ☐ No →
- ☐ Yes

Did you use a contraception at this time?
- ☐ No → **go to page 9**

What kind of contraception?
- ☐ Condom
- ☐ Pill
- ☐ Emergency pill
- ☐ Withdraw
- ☐ Other: ....

SIGNIFICANT OTHERS

You share affection and support with different persons

In general, with whom do most share these types of moments?

(they can be family members or others)

	Never	*Relationship (for exemple uncle, older sister/brother. Don't give their name)*	*Nobody*
I laugh and exchange jokes with	☐	*1..............................* *2..............................*	☐
I get cuddled by	☐	*1..............................* *2..............................*	☐
I chat over my affairs, boy-friend stories with	☐	*1..............................* *2..............................*	☐
When I have nightmares or frightening thoughts, I get comforted by	☐	*1..............................* *2..............................*	☐
When I am sick, I turn to	☐	*1..............................* *2..............................*	☐
When I have problems at school or work, I discuss with	☐	*1..............................* *2..............................*	☐
When I have money problems, I discuss issues with	☐	*1..............................* *2..............................*	☐
I get information about my health, illness or medication from	☐	*1..............................* *2..............................*	☐
If I do something wrong, I can confide in	☐	*1..............................* *2..............................*	☐
If somebody does something bad to me, I can confide in	☐	*1..............................* *2..............................*	☐
I discuss about the future with	☐	*1..............................* *2..............................*	☐

RELATIONSHIP WITH ADULTS

How easy/difficult is it to ask any questions to the people at the hospital (nurses and doctors)?

- ☐ Very Easy
- ☐ Easy
- ☐ OK
- ☐ Difficult
- ☐ Very difficult

In the past year, did you have arguments with your caregiver or parents?

- ☐ Very often
- ☐ Often
- ☐ Sometimes
- ☐ Rarely
- ☐ Never

YOUR PERSONALITY

Do you think that you are generally

- ☐ Joyful
- ☐ Intermediate
- ☐ Sad
- ☐ Up and down

Do you think that you are generally

- ☐ Lonely
- ☐ Sociable/Friendly

Do you think that you are generally

- ☐ Always obeisant
- ☐ Sometimes obeisant

How do you generally feel?

- ☐ Very unhappy
- ☐ Unhappy
- ☐ Average
- ☐ Happy
- ☐ Very happy

Do you have a special friend with whom you feel very close?

- ☐ No
- ☐ Yes ☐ boy ☐ girl

Do you meet often? ☐ Yes ☐ No

Do you chat together on the internet or mobile phone? ☐ Yes ☐ No

PEER GROUP

Do you have a group of kids with whom you spend time?
☐ No
☐ Yes

Where do they mostly come from?
☐ School
☐ Neighborhood
☐ Family
☐ Others

Are they
☐ Mostly boys
☐ Mostly girls
☐ Equally boys and girls

What do you like to do most with your group of peers? ……………………………

GIRL/BOY FRIENDS/FAVORITE

Do you have a boy/girl friend?
☐ No → GO TO PAGE 12
☐ Yes ☐ boy ☐ girl

Since you were |__|__| years old

Do you have sex together?
☐ No → GO TO PAGE 12
☐ Yes

The last time you had sex, did you use condom?
☐ No
☐ Yes

Do you take contraception
☐ No → GO TO PAGE 12
☐ Yes

What kind of contraception?
☐ Condom
☐ Pill
☐ Emergency pill
☐ Injectable
☐ Other: …………………………

Have you ever been pregnant?
☐ 2 No → GO TO PAGE 12
☐ 1 Yes

If yes, how old were **you**? |__|__| years old

If yes, how old was your partner? |__|__| years old

Did you
☐ continue with the pregnancy
☐ have an abortion

ACTIVITIES

Do you play computer games?	☐ No<br>☐ Sometimes<br>☐ Regularly
Do you smoke cigarettes?	☐ No<br>☐ Sometimes<br>☐ Regularly
Do you like riding motorbike very fast?	☐ Don’t know how to ride<br>☐ No<br>☐ Yes
Have you ever had a motorbike accident while you were driving?	☐ No<br>☐ Yes
Do you spend time chatting on the internet?	☐ No<br>☐ Sometimes<br>☐ Often
Do you listen to music?	☐ No<br>☐ Sometimes<br>☐ Often
Do you read books?	☐ No<br>☐ Sometimes<br>☐ Often
Do you drink energizing drinks, like *Red-Bull* ?	☐ No<br>☐ Sometimes<br>☐ Often
Do you drink beer?	☐ No<br>☐ Sometimes<br>☐ Often

Do you drink strong alcohol such as *Sangsom*?	☐ No<br>☐ Sometimes<br>☐ Often
Have you ever been drunk?	☐ No<br>☐ Once<br>☐ Sometimes<br>☐ Often
Do you hang out with your friends?	☐ No<br>☐ Sometimes<br>☐ Often
Where do you hang out?	☐ At home<br>☐ At my friends' house<br>☐ At the mall<br>☐ In the bar, restaurant, etc.<br>☐ Outside such as parks, streets, stadium, etc…<br>☐ Other: ....
Do you know people who take drugs?	☐ No<br>☐ Yes
Do you practice a sport	☐ No<br>☐ Sometimes<br>☐ Regularly
Which sport?	……………………………………………..
Do you watch sport on TV?	☐ No<br>☐ Sometimes<br>☐ Often
Was is your favorite sport?	………………………………………………
Do you have a mobile phone?	☐ Yes ☐ No

Do you watch soap operas on TV?	☐ No<br>☐ Sometimes<br>☐ Often
What is your favorite soap series?	....
Do you do gardening?	☐ No<br>☐ Sometimes<br>☐ Often
Do you do some cooking?	☐ No<br>☐ Sometimes<br>☐ Often
Do you (have you) attend(ed) extra-curriculum school	☐ Yes<br>☐ No
What is your religion?	☐ I do not have a religion<br>☐ Buddhist<br>☐ Christian<br>☐ Muslim<br>☐ Other (specify: ....)
When did you last go to the Wat/Church/Mosque/Other?	☐ Last week<br>☐ Last month<br>☐ Last 3 months<br>☐ Last year<br>☐ For festivals only<br>☐ Not for a long time

THANK YOU VERY MUCH FOR YOUR HELP !
