# Supplementary material for: Factors influencing antiretroviral treatment suboptimal adherence among perinatally HIV-infected adolescents in Thailand
Source: PLoS One. 2017 Feb 16;12(2):e0172392. doi: 10.1371/journal.pone.0172392 (PMC5312953; doi:10.1371/journal.pone.0172392)
Supplement: S2 File — (PDF) [file pone.0172392.s002.pdf]

**สถาบันการศึกษาประชากรแห่งชาติ ปารีส**

**สถาบันวิจัยสังคม มหาวิทยาลัยเชียงใหม่ จังหวัดเชียงใหม่**

# แบบสอบถามสำหรับวัยรุ่น

**พฤศจิกายน 2552**

**เด็กผู้หญิง**

ตอบแบบสอบถามด้วยตนเอง

หมายเลขประจำตัว: [ ] HN :......................................

วันที่: ..................................... |___|___| |___|___| |2|5|___|___|

วันที่ เดือน พ.ศ.

***โปรดแนะนำโครงการวิจัยและสอบถามเพื่อขอความยินยอม***

สามารถกรอกเองได้: ☐ ต้องช่วยกรอก: ☐

สถานที่วิจัย: ............................................ รหัส : |__|__|

# ถ้าหนูอาศัยอยู่กับครอบครัว / อยู่บ้านส่วนตัว ให้กรอกข้อมูลหน้านี้

ปัจจุบันหนูอายุเท่าไหร่? |____|____|ปี

หนูเกิดวันที่เท่าไหร่ (ปีที่หนูเกิด) |____|____| |____|____| |2|5|____|____|

วันที่ เดือน พ.ศ.

หนูอาศัยอยู่กับใครบ้างที่บ้าน? อยู่คนเดียว ☐ → ข้ามไปหน้า **4**

| ระบุความสัมพันธ์ เช่น พ่อแม่ ปู่ย่า ตายาย พี่น้อง ญาติ ฯลฯ | หนูนอนห้องเดียวกับเขาไหม? | หนูรู้สึกสนิทกับเขาไหม? |
|---|---|---|
| 1. ........................................ | ☐ | ☐ |
| 2. ........................................ | ☐ | ☐ |
| 3. ........................................ | ☐ | ☐ |
| 4. ........................................ | ☐ | ☐ |
| 5. ........................................ | ☐ | ☐ |
| 6. ........................................ | ☐ | ☐ |
| 7. ........................................ | ☐ | ☐ |
| 8. ........................................ | ☐ | ☐ |

ผู้ที่ดูแลหนูใกล้ชิดมากที่สุดที่บ้านคือใคร................................

หนูช่วยงานบ้านหรือเปล่า (เช่น ช่วยทำความสะอาด ช่วยซื้อของ ช่วยทำอาหาร หรือ อื่นๆ) ?

☐ ช่วยเป็นประจำ
☐ ช่วยเป็นบางครั้ง
☐ ช่วยบ้างเล็กน้อย
☐ ไม่เคยช่วยเลย

หนูมีงานบ้านที่ต้องรับผิดชอบหรือเปล่า? (เช่น รดน้ำต้นไม้ ล้างจาน หรืออื่นๆ)

☐ ไม่มี
☐ มี งานอะไร?.............

หนูได้ช่วยผู้ใหญ่ทำงานอะไรเป็นประจำหรือเปล่า? (ในร้าน ในไร่ นา สวน หรืออื่นๆ)

☐ ไม่มี
☐ มี งานอะไร?.............

หนูต้องดูแลใครในบ้านเป็นประจำหรือเปล่า

☐ ไม่มี
☐ มี ใคร?.....................

## ที่โรงเรียน

ปัจจุบันนี้หนูเรียนหนังสืออยู่หรือไม่ ☐ เรียน ชั้นไหน..................
☐ ไม่เรียน ได้เรียนสูงสุดถึงชั้นไหน..............................

หนูชอบเรียนหนังสือไหม?
- ☐ ชอบมาก
- ☐ ชอบนิดหน่อย
- ☐ เฉยๆ
- ☐ ไม่ชอบ
- ☐ ไม่ชอบเลย

หนูออกจากโรงเรียนหรือเคยหยุดเรียนเพราะหนูป่วยหรือเปล่า
- ☐ ใช่ เป็นประจำ
- ☐ ใช่ นานมาแล้ว
- ☐ บางครั้ง
- ☐ นานๆจะมีครั้งหนึ่ง

ผลการเรียนของหนูเป็นอย่างไร ?
- ☐ ดีมาก
- ☐ ดี
- ☐ ปานกลาง
- ☐ แย่
- ☐ แย่มาก

หนูมีเพื่อนที่โรงเรียนไหม?
- ☐ มีเยอะมาก
- ☐ มากกว่า 3 คน
- ☐ มี 2-3 คน
- ☐ ไม่มีเลย

## ที่ทำงาน

หนูเคยทำงานที่ได้รับค่าจ้างบ้างหรือไม่?

☐ ไม่ (ข้ามไปตอบตามลูกศร) ☐ ใช่

หนูทำงานอะไร? ......................................

หนูทำงานสัปดาห์ละกี่วัน? |___|

หนูได้เงินเท่าไหร่? ถ้าได้เป็นรายวัน |___|___|___| *ต่อวัน*

*ถ้าได้เป็นรายเดือน* |___|___|___|___| *ต่อ เดือน*

หนูให้เงินช่วยเหลือค่าใช้จ่ายภายในบ้านบ้างหรือเปล่า? ☐ ไม่ ☐ บางครั้ง ☐ เป็นประจำ

หนูชอบงานที่หนูทำหรือเปล่า? ☐ ไม่ ☐ ใช่

หนูทำงานเหนื่อยไหม? ☐ ไม่ ☐ ใช่

หนูมีเพื่อนที่ทำงานหรือเปล่า? ☐ มีมาก ☐ มีพอสมควร ☐ มีน้อย ☐ ไม่มีเลย

## อนาคต

หนูวางแผนเรียนต่อในระดับมหาวิทยาลัย/วิทยาลัย/อาชีวศึกษา หรือไม่? ☐ ไม่ ☐ ใช่ ☐ ไม่ทราบ

หนูต้องการเรียนเกี่ยวกับอะไร? ............................................... ☐ ไม่ทราบ

อาชีพที่หนูใฝ่ฝันคืออะไรไ ................................................... ☐ ไม่ทราบ

ในอนาคตหนูคิดว่าจะแต่งงานหรือมีคู่ครองหรือไม่ ☐ ใช่ ☐ ไม่ ☐ หนูไม่แน่ใจ

ในอนาคต หนูต้องการที่จะมีลูกหรือไม่? ☐ ใช่ ☐ ไม่ ☐ หนูไม่แน่ใจ

เมื่อหนูโตขึ้นหนูคิดที่จะย้ายไปอยู่ที่อื่นหรือไม่ ☐ ใช่ ☐ ไม่ ☐ ไม่แน่ใจ

## สุขภาพ

ตอนนี้สุขภาพของหนูเป็นยังไงบ้าง? ☐ ดีมาก
☐ ดี
☐ ปานกลาง
☐ แย่
☐ แย่มาก

เมื่อเทียบกับเพื่อนๆ หนูเป็นอย่างไร

| **ส่วนสูง** | **น้ำหนัก** |
| --- | --- |
| ☐ สูงกว่า | ☐ อ้วนกว่า |
| ☐ ตามเกณฑ์เฉลี่ย | ☐ ตามเกณฑ์เฉลี่ย |
| ☐ เตี้ยกว่า | ☐ ผอมกว่า |

หนูเคยนอนโรงพยาบาลหรือไม่ ☐ ไม่เคย (ข้ามไปตอบตามลูกศร)
☐ เคย

ตอนที่หนูเข้าโรงพยาบาลครั้งล่าสุด หนูอายุเท่าไหร่?|___|___|ปี

หนูอยู่โรงพยาบาลนานแค่ไหน? |___|___| เดือน |___|___|วัน

หนูกินยาหรือได้รับการรักษาใดๆอย่างประจำสม่ำเสมอหรือไม่? ☐ ไม่ → **ไปหน้าที่ 8**
☐ ใช่

หนูเริ่มกินยา เมื่ออายุเท่าไหร่? |___|___| ปี ☐ จำไม่ได้

หนูมีผลข้างเคียงจากการกินยาหรือไม่ ☐ ไม่
☐ ใช่ (ระบุ): ................................

มีใครช่วยเอายาให้หนูกินไหม? ☐ ไม่ หนูกินยาเอง
☐ มี ใคร? ................................................

มีใครคอยเตือนให้หนูกินยาไหม? ☐ ไม่มี
☐ มี เป็นบางครั้ง ใคร?..........................
☐ มีคนคอยเตือนเป็นประจำ ใคร?..........................

| | |
|---|---|
| หนูคิดว่าเป็นเรื่องง่ายหรือยากในการจำว่าต้องกินยา? | ☐ ง่ายมาก<br>☐ ง่าย<br>☐ ธรรมดา<br>☐ ยาก<br>☐ ยากมาก |
| หนูใช้วิธีใดในการเตรียมหรือเตือนความจำเพื่อให้หนูกินยาได้ตรงเวลา?<br>(ทำเครื่องหมายถูกหน้าคำตอบได้มากกว่า 1 ข้อ) | ☐ ไม่มี<br>☐ ใช้ดูเวลาจากนาฬิกา<br>☐ ใช้เสียงเตือนจากนาฬิกาปลุก<br>☐ ใช้เสียงเตือนจากโทรศัพท์มือถือ<br>☐ ใช้เสียงเพลงชาติ<br>☐ ใช้เสียงเตือนจากรายการโทรทัศน์ / วิทยุ<br>☐ ใช้กล่องยาที่มีช่องแบ่งยา<br>☐ อื่นๆ: ........................................ |
| ช่วงสัปดาห์ที่แล้ว (7 วัน) หนูเคยลืมกินยาหรือเปล่า? | ☐ ไม่เคย<br>☐ เคย 1 ครั้ง<br>☐ เคยหลายครั้ง<br>☐ ไม่รู้ |
| เมื่อหนูลืมกินยา อะไรเป็นเหตุผลสำคัญที่ทำให้ลืม ? | ☐ ป่วย<br>☐ เล่นจนเพลิน<br>☐ ยุ่งมาก<br>☐ รู้สึกเบื่อหน่าย<br>☐ ไม่อยู่บ้าน<br>☐ เป็นช่วงเทศกาล / วันหยุด<br>☐ ไม่มีใครเอายาให้หนูกิน<br>☐ ไม่มีความเป็นส่วนตัวเพียงพอ/กลัวว่าคนอื่นจะเห็น<br>☐ เหตุผลอื่นๆ ระบุ:…………………………………………….<br>……………………………………………………………………….. |
| เวลาที่หนูลืมกินยาหนูเคยโดนตำหนิหรือเปล่า?<br>(เช่น จากพยาบาล / หมอ /สมาชิกในครอบครัว) | ☐ เคย<br>☐ ไม่เคย |

**อนามัยเจริญพันธุ์**

หนูมีประจำเดือนหรือยัง?
- ☐ ยังไม่มี
- ☐ มี เมื่ออายุ |__|__| ปี

หนูเคยได้รับความรู้หรือได้รับการอบรมเรื่องเพศศึกษา/การวางแผนครอบครัวหรือไม่?
- ☐ ไม่เคย
- ☐ เคย

ถ้าเคยได้รับการอบรม จากที่ไหน
- ☐ ที่โรงเรียน
- ☐ ในค่ายของเยาวชน
- ☐ ที่โรงพยาบาล
- ☐ ที่บ้าน
- ☐ อื่นๆ ระบุ: ........................

หนูเคยมีเพศสัมพันธ์หรือเปล่า?
- ☐ ไม่ขอตอบคำถามนี้ → ไปหน้าที่ 9
- ☐ ไม่ เคย → ไปหน้าที่ 9
- ☐ เคย ครั้งแรกเมื่ออายุ |__|__| ปี

↓

กับใคร?
- ☐ คู่ครอง
- ☐ หญิงขายบริการ
- ☐ แฟน
- ☐ ชายขายบริการ
- ☐ คู่นอนชั่วคราว
- ☐ ถูกข่มขืน
- ☐ อื่นๆ (ระบุ): ..........................................................................................

..........................................................................................

อายุของคู่นอนในการมีเพศสัมพันธ์ครั้งแรก |__|__| ปี ☐ ไม่ทราบ

หนูใช้ถุงยางอนามัยในเพศสัมพันธ์ครั้งแรกหรือเปล่า?
- ☐ ไม่ใช้
- ☐ ใช้

หนูใช้วิธีการคุมกำเนิดในเพศสัมพันธ์ครั้งแรกหรือเปล่า?
- ☐ ไม่ใช้ → *ไปหน้าที่ 9*
- ☐ ใช้

หนูใช้การคุมกำเนิดวิธีไหนในการมีเพศสัมพันธ์ครั้งแรก?
- ☐ ถุงยางอนามัย
- ☐ ยาเม็ดคุมกำเนิด
- ☐ ยาเม็ดคุมกำเนิดแบบฉุกเฉิน
- ☐ หลั่งข้างนอก
- ☐ อื่นๆ: ...............................

## คำถามอื่น ๆ ที่สำคัญ

การแบ่งปันความรักและการได้รับการช่วยเหลือ

| โดยปกติแล้วหนูได้แบ่งปันช่วงเวลาสำคัญเหล่านี้กับใครมากที่สุด? (อาจเป็นสมาชิกในครอบครัว หรือผู้อื่นก็ได้) | ไม่เคยเกิดขึ้น | ความสัมพันธ์ (เช่น พี่ ป้า น้า อา) ไม่ต้องระบุชื่อ | ไม่มีใคร |
|---|---|---|---|
| หนูหัวเราะและพูดคุยเรื่องตลกกับ | ☐ | 1................................<br>2................................ | ☐ |
| หนูได้รับการกอดโดย | ☐ | 1................................<br>2................................ | ☐ |
| หนูพูดคุยเรื่องแฟน เรื่องความรัก ของหนูกับ | ☐ | 1................................<br>2................................ | ☐ |
| เวลาฝันร้ายหรือมีความกลัวเกิดขึ้น หนูได้รับการปลอบโยนจาก | ☐ | 1................................<br>2................................ | ☐ |
| เวลาหนูป่วย หนูขอความช่วยเหลือจาก | ☐ | 1................................<br>2................................ | ☐ |
| เวลาหนูมีปัญหาที่โรงเรียนหรือปัญหาด้านการทำงาน หนูปรึกษากับ | ☐ | 1................................<br>2................................ | ☐ |
| เวลาหนูมีปัญหาเรื่องเงิน หนูปรึกษากับ | ☐ | 1................................<br>2................................ | ☐ |
| หนูได้รับข้อมูลเกี่ยวกับเรื่องการเจ็บป่วยของหนูหรือเรื่องยาจาก | ☐ | 1................................ | ☐ |

| | | | |
|---|---|---|---|
| | | 2................................ | |
| เมื่อหนูทำบางสิ่งบางอย่างผิดพลาด หนูสามารถบอกความลับนี้กับ | ☐ | 1................................<br>2................................ | ☐ |
| เมื่อมีคนทำสิ่งไม่ดีกับหนู หนูสามารถบอกเรื่องนี้กับ | ☐ | 1................................<br>2................................ | ☐ |
| หนูปรึกษาเรื่องอนาคตกับ | ☐ | 1................................<br>2................................ | ☐ |

## ความสัมพันธ์กับเจ้าหน้าที่โรงพยาบาล

| | |
|---|---|
| การพูดคุยกับเจ้าหน้าที่โรงพยาบาล (เช่นพยาบาล และแพทย์) มีความง่าย หรือยากอย่างไร? | ☐ ง่ายมาก<br>☐ ง่าย<br>☐ ปานกลาง<br>☐ ยาก<br>☐ ยากมาก |

## ความสัมพันธ์กับผู้ใหญ่

การพูดคุยกับพ่อแม่ผู้ปกครองมีความง่าย หรือยากอย่างไร?
- ☐ ง่ายมาก
- ☐ ง่าย
- ☐ ปานกลาง
- ☐ ยาก
- ☐ ยากมาก

ในปีที่ผ่านมา หนูได้มีการโต้เถียงกับผู้ดูแล หรือพ่อแม่ของหนูหรือไม่?
- ☐ บ่อยมาก
- ☐ บ่อย
- ☐ บางครั้ง
- ☐ นาน ๆ ครั้ง
- ☐ ไม่เคย

## บุคลิกภาพของหนู

โดยทั่วไปหนูคิดว่าหนูเป็นคน
- ☐ ร่าเริง
- ☐ เฉยๆ
- ☐ เศร้า
- ☐ อารมณ์เปลี่ยนแปลงง่าย

โดยทั่วไปหนูคิดว่าหนูเป็นคน
- ☐ ชอบอยู่คนเดียว เก็บตัว
- ☐ ชอบเข้าสังคม มีมนุษยสัมพันธ์ดี

โดยทั่วไปหนูคิดว่าหนูเป็นคน
- ☐ เชื่อฟังผู้ใหญ่เสมอ
- ☐ เชื่อฟังผู้ใหญ่เป็นบางครั้ง

โดยทั่วไปหนูรู้สึกอย่างไร
- ☐ ไม่มีความสุขเลย
- ☐ มีความสุขเล็กน้อย
- ☐ มีความสุขปานกลาง
- ☐ มีความสุขมาก
- ☐ มีความสุขมากที่สุด

หนูมีเพื่อนสนิทหรือไม่?
- ☐ ไม่มี
- ☐ มี ☐ เป็นผู้ชาย ☐ เป็นผู้หญิง

หนูกับเพื่อนสนิทพบกันบ่อยหรือไม่? ☐ ใช่ ☐ ไม่ใช่

หนูได้คุย (แช็ท) กับเพื่อนสนิททางอินเตอร์เน็ตหรือทางมือถือหรือไม่? ☐ ใช่ ☐ ไม่ใช่

## เพื่อนในกลุ่ม

หนูได้ใช้เวลาอยู่กับกลุ่มเพื่อนๆหรือไม่ ☐ ไม่ใช่
☐ ใช่

กลุ่มเพื่อนส่วนใหญ่เป็นเพื่อนจากที่ใด
☐ จากโรงเรียน
☐ เพื่อนแถวบ้าน
☐ ญาติพี่น้องรุ่นเดียวกัน
☐ อื่น ระบุ..........................

เพื่อนในกลุ่มส่วนใหญ่เป็นเพศใด
☐ ส่วนมากเป็นผู้ชาย
☐ ส่วนมากเป็นผู้หญิง
☐ ผู้ชายและหญิงเท่าๆ กัน

ส่วนใหญ่แล้วชอบทำกิจกรรมใดมากที่สุดในกลุ่มเพื่อน ..........................................

## แฟน

หนูมีแฟนหรือยัง? ☐ ไม่มี → *ไปหน้าที่ 12*
☐ มี เป็น ☐ ผู้ชาย ☐ ผู้หญิง

หนูมีแฟนตั้งแต่อายุ |__|__| ปี

หนูมีเพศสัมพันธ์กับแฟนหรือไม่? ☐ ไม่มี → *ไปหน้าที่ 12*
☐ มี

ครั้งล่าสุดที่มีเพศสัมพันธ์กัน หนูได้ใช้การคุมกำเนิดใดๆหรือไม่? ☐ ไม่ → *ไปหน้าที่ 12*
☐ ใช่

หนูใช้วิธีการคุมกำเนิดอะไร?
☐ ถุงยางอนามัย
☐ ยาเม็ดคุมกำเนิดแบบฉุกเฉิน
☐ ยาเม็ดคุมกำเนิด
☐ ยาคุมกำเนิดชนิดฉีด
☐ อื่น ๆ: ..............................

หนูเคยตั้งท้องหรือไม่? ☐ ไม่ → *ไปหน้าที่ 12*
☐ ใช่

ถ้าเคย ตอนนั้น**หนู**อายุเท่าไหร่? |__|__| ปี

ถ้าเคย ตอนนั้นแฟนหนูอายุเท่าไหร่? |__|__| ปี

การตั้งครรภ์นั้นเป็นอย่างไร
☐ ตั้งครรภ์จนคลอด
☐ ทำแท้ง

## กิจกรรม

| | |
|---|---|
| หนูเล่นเกมส์คอมพิวเตอร์หรือไม่? | ☐ ไม่<br>☐ บางครั้ง<br>☐ เป็นประจำ |
| หนูสูบบุหรี่หรือไม่? | ☐ ไม่<br>☐ บางครั้ง<br>☐ เป็นประจำ |
| หนูชอบการขี่รถมอเตอร์ไซค์เร็วๆ หรือไม่ ? | ☐ ไม่ชอบ<br>☐ ชอบ<br>☐ ขี่ไม่เป็น |
| หนูเคยได้รับอุบัติเหตุในขณะที่ขี่รถมอเตอร์ไซค์หรือไม่? | ☐ ไม่<br>☐ ใช่ |
| หนูได้ใช้เวลาในการแชททางอินเตอร์เนตหรือไม่? | ☐ ไม่<br>☐ บางครั้ง<br>☐ บ่อยครั้ง |
| หนูฟังเพลงหรือไม่? | ☐ ไม่<br>☐ บางครั้ง<br>☐ บ่อยครั้ง |
| หนูอ่านหนังสือหรือไม่? | ☐ ไม่<br>☐ บางครั้ง<br>☐ บ่อยครั้ง |
| หนูดื่มเครื่องดื่มชูกำลัง เช่น กระทิงแดง ลิโพ หรือไม่? | ☐ ไม่<br>☐ บางครั้ง<br>☐ บ่อยครั้ง |
| หนูดื่มเบียร์หรือไม่? | ☐ ไม่<br>☐ บางครั้ง<br>☐ บ่อยครั้ง |

หนูดื่มแอลกอฮอล์แรงๆ เช่น รีเจนซี่ , เหล้าขาว หรือไม่?
- ☐ ไม่
- ☐ บางครั้ง
- ☐ บ่อยครั้ง

หนูเคยเมาไหม?
- ☐ ไม่เลย
- ☐ หนึ่งครั้ง
- ☐ บางครั้ง
- ☐ บ่อยครั้ง

หนูออกไปสังสรรค์กับเพื่อนของหนูหรือไม่?
- ☐ ไม่
- ☐ บางครั้ง
- ☐ บ่อยครั้ง

หนูไปสังสรรค์ที่ไหน?
- ☐ ที่บ้านตนเอง
- ☐ ที่บ้านเพื่อน
- ☐ ที่ห้างสรรพสินค้า
- ☐ ในผับ/บาร์/ร้านอาหาร/ร้านเหล้าตอง
- ☐ ที่สาธารณะ เช่น สวนสาธารณะ สถานที่ท่องเที่ยว สนามกีฬา
- ☐ ที่อื่น ๆ: ................................

หนูรู้จักคนที่ใช้ยาเสพติดหรือไม่?
- ☐ ไม่รู้จัก
- ☐ รู้จัก

หนูเล่นกีฬาหรือไม่?
- ☐ ไม่
- ☐ บางครั้ง
- ☐ เป็นประจำ

หนูเล่นกีฬาอะไร? ..................................................

หนูดูกีฬาทางทีวีหรือไม่?
- ☐ ไม่
- ☐ บางครั้ง
- ☐ บ่อยครั้ง

กีฬาที่หนูชื่นชอบคืออะไร? ..................................................

หนูมีโทรศัพท์มือถือมั๊ย? ☐ มี ☐ ไม่มี

หนูดูละครทีวีหรือไม่?
- ☐ ไม่
- ☐ บางครั้ง
- ☐ บ่อยครั้ง

หนูชอบดูละครเรื่องอะไร? ..........................................................

หนูปลูกต้นไม้หรือไม่?
- ☐ ไม่
- ☐ บางครั้ง
- ☐ บ่อยครั้ง

หนูทำอาหารหรือไม่?
- ☐ ไม่
- ☐ บางครั้ง
- ☐ บ่อยครั้ง

หนูกำลังเรียนหรือเคยเรียนพิเศษหรือไม่?
- ☐ ใช่
- ☐ ไม่

หนูนับถือศาสนาอะไร?
- ☐ ไม่ได้นับถือศาสนาใด
- ☐ พุทธ
- ☐ คริสต์
- ☐ อิสลาม
- ☐ อื่น ๆ (ระบุ : ..........................)

หนูไปวัด/โบสถ์/ สุเหร่า/ อื่น ๆ ครั้งสุดท้ายเมื่อไหร่?
- ☐ สัปดาห์ที่แล้ว
- ☐ เดือนที่แล้ว
- ☐ 3 เดือนที่แล้ว
- ☐ ปีที่แล้ว
- ☐ ช่วงเทศกาลเท่านั้น
- ☐ ไม่ได้ไปเป็นเวลานาน

ขอบคุณมากสำหรับความร่วมมือ !
