# Supplementary material for: Factors influencing antiretroviral treatment suboptimal adherence among perinatally HIV-infected adolescents in Thailand
Source: PLoS One. 2017 Feb 16;12(2):e0172392. doi: 10.1371/journal.pone.0172392 (PMC5312953; doi:10.1371/journal.pone.0172392)
Supplement: S3 File — (PDF) [file pone.0172392.s003.pdf]

Institut National d'Etudes Démographiques, Paris
Social Research Institute, Chiang Mai University, Chiang Mai

PARENT / GUARDIAN QUESTIONNAIRE

April 2010

ID Number: [ ]

HN Number of the child: ....

Child

Sex of child you are responsible of: ☐ 1 Male ☐ 2 Female

Interview date: |__|__| day |__|__| month |_2_|_5_|_5_|_ _| yr

Time interview begun: |__|__| Hour |__|__| Minutes

Please introduce the study and ask for consent

Interviewer's Name : .... Code: |__|__|

Site: .... Code : |__|__|

SECTION R: RESPONDENT

We would like first to know about you, as the caregiver of the child			
R.0	Sex	☐ 1 Male<br>☐ 2 Female	
R.00	Age of respondent	|__|__| years	
R.1	What is your relationship with the child?	……………………………………………………………….	|__|__|
R.2	What is your marital status?	☐ 1 Single<br>☐ 2 Married<br>☐ 3 Divorced/separated<br>☐ 4 Widowed	
G.1	What is your nationality?	☐ 1 Thai citizen<br>☐ 2 Non Thai, specify……………………………	|__|__|
G.2	What is your ethnicity?	☐ 1 Thai<br>☐ 2 Others, specify………………………………	|__|__|
R.3	Have you ever attended school?<br><br>Comments:………………………………………………………	☐ 1 Yes<br>☐ 2 No	⇒**Q** R.5
R.4	What was your education level when you finished school	Highest Grade level: |__|__| <br>☐ 1 Primary<br>☐ 2 Secondary<br>☐ 3 Teacher college<br>☐ 4 Vocational college<br>☐ 5 University<br>☐ 6 Other (specify)……………………….	
R.5	What is your current occupation?	…………………………………………….	|__|__|
R.6	How would you rate your health status situation<br><br>*REFER TO THE "SMILE"* ***SCALE***<br><br>Comments: …………………………………………………..	☐ 1 Very poor<br>☐ 2 poor<br>☐ 3 Fair<br>☐ 4 good<br>☐ 5 Very good	|__|__|
R.7	Do you have any chronic disease (including HIV infection) or permanent disability?	☐ 1 No<br>☐ 2 Yes, specify: ……………………..	

SECTION A: THE CHILD'S LIFE

NO.	QUESTIONS & FILTERS	CODING CATEGORIES	GO TO

CHILD:

	Now we are going to ask you some questions about the child you are responsible for		
**A.1.**	**Age (column 2)**<br>How old is the child ?<br>*CIRCLE THE AGE OF THE CHILD*	|___|___|years old today	***Column 2***<br>*Age*
**A.2**	**Years (Column 1)**<br>What is his/her date of birth (year that he/she was born)	|___|___| |___|___| |_2_|_5_|___|___| DAY MONTH YEAR	***Column 1***<br>*Years*
	1. COMPLETE THE YEAR OF BIRTH AND ALL THE YEARS UNTIL THE PRESENT YEAR.<br>*2. DRAW A LINE FOR THE PRESENT YEAR*		

G.3	What is the child's nationality?	☐ 1 Thai citizen<br>☐ 2 Non Thai specify………………………	|__|__|
G.4	What is the child's ethnicity?	…………………………………………………………	|__|__|

Situation of the child's biological parents

**MOTHER**			
B.3	***Only if the child's mother is not the respondent, ask the following question***<br><br>Is the child's mother alive?	☐ 1 Yes<br>☐ 10 Live in the household ➔**B.6**<br>☐ 11 Do not live in the household➔**B.6**<br>***Note that you will have to complete Section BB next page***<br>☐ 3 No ☐ 98 Don't know	
B.4	*WRITE* **MDTH** *IN THE COLUMN PARENTS STATUS (COLUMN 3) AT THE AGE OF THE CHILD WHEN THE MOTHER DIED* **MDTH** |___|___|years or |___|___|age		***Column 3***
B.5	If the mother has died, was the death related to HIV/AIDS?	☐ 1 Yes, comments: ……………………………………<br>☐ 2 No, Comments: ……………………………………<br>☐ 98 Don't know	
**FATHER**			
B.6	***Only if the child's father is not the respondent, ask the following question***<br><br>Is the child's father alive?	☐ 1 Yes<br>☐ 10 Live in the household ➔**B.9**<br>☐ 11 Do not live in the household➔**B.9**<br>***Note that you will have to complete Section BB next page***<br>☐ 3 No ☐ 98 Don't know	
B.7	*WRITE* **FDTH** *IN THE COLUMN PARENTS STATUS (COLUMN 3) AT THE AGE OF THE CHILD WHEN THE FATHER DIED* **FDTH** |___|___|years or |___|___|age		***Column 3***
B.8a	If the father has died, was the death related to HIV/AIDS?	☐ 1 Yes, comments: ……………………………………<br>☐ 2 No, Comments: ……………………………………<br>☐ 98 Don't know	
B.8b	*IF THE PARENTS GOT SEPARATED, WRITE MF-SEP THE YEAR THEY SEPARATED*<br>**FM-SEP** |___|___|years or |___|___|age		***Column 3***
**SIBLINGS** (***only from same father and mother***)			
B.9	How many siblings has the child including those who have died?<br>*If other siblings (not same father and mother) are mentioned, specify*: ……………………………….<br>…………………………………………………………………….	|__|__| *including the child*	
B.10	How many are still alive?	|__|__| ☐ 98 Don't know	
B.11	How many siblings are infected with HIV?	|__|__| ☐ 98 Don't know	
B.12	How many siblings died of HIV/AIDS?	|__|__| ☐ 98 Don't know	

CARE-GIVERS OF THE CHILD

B.13	**Life Events Table: caregivers (Column 4)**<br>*LIST THE CHILD'S CAREGIVERS SINCE BIRTH (RELATIONSHIP WITH THE CHILD IN FULL)*	***Column 4***<br>*Caregiver*

HOUSEHOLD OF THE CHILD

I will ask you now about the persons living with the child

	Household Form	Co-residents	House-hold Form
H.1	1. *FILL IN THE **PART H** OF THE HOUSEHOLD FORM:*<br>**2.** *LIST THE CO-RESIDENTS ONE BY ONE, ASK THEIR AGE, **THEIR RELATIONSHIP WITH THE CHILD**, AS WELL AS THEIR OCCUPATION*		

Section BB ***This section only applies if the mother is alive and is not living in the household or if the father is alive and is not living in the household***

	Father's status	Mother's status
BB.1	Is the father HIV infected?<br>☐ 1 Yes<br>☐ 2 No<br>☐ 98 Don't know	Is the mother HIV infected?<br>☐ 1 Yes<br>☐ 2 No<br>☐ 98 Don't know
BB.2	Where does he lives?...........................	Where does she lives?....................................
BB.3	**W**hat is his occupation?.........................	**W**hat is her occupation?................................
BB.4	**D**oes he send money for the child care<br>☐ 1 Yes<br>☐ 2 No	**D**oes she send money for the child care<br>☐ 1 Yes<br>☐ 2 No
BB.5	What is the father's family situation<br>☐ 1 Still Married<br>☐ 2 Separated<br>☐ 3 Remarried,<br>☐ 4 Remarried with other children<br>☐ 5 Other…………………………………………..<br>☐ 98 Don't know	What is the mother's family situation<br>☐ 1 Still Married<br>☐ 2 Separated<br>☐ 3 Remarried,<br>☐ 4 Remarried with other children<br>☐ 5 Other…………………………………………………..<br>☐ 98 Don't know
BB.6	How often the child has seen his/her father **<u>in the past year</u>**?<br>☐ 1 More than once a week ....<br>☐ 2 A few times a month....<br>☐ 3 A few times a year ....<br>☐ 4 Once a year or less.................<br>☐ 5 Never ....<br>☐ 6 Other (specify):<br>(*Only one answer possible*)	How often has the child has seen his/her mother **<u>in the past year</u>**?<br>☐ 1 More than once a week ....<br>☐ 2 A few times a month....<br>☐ 3 A few times a year ....<br>☐ 4 Once a year or less.................<br>☐ 5 Never ....<br>☐ 6 Other (specify):<br>(*Only one answer possible*)

PLACES OF RESIDENCE OF THE CHILD

	I want to talk with you about the places where the child has lived for more than one year throughout his/her life	
A.3	**Places of Residence (Column 5)**<br>Where (town/village) have they been living since birth and when?<br><br>*FILL IN ALL THE PLACES OF RESIDENCE (**town/village**) EVER LIVED FOR AT LEAST ONE YEAR SINCE BIRTH UNTIL THE PRESENT YEAR*	***Column 5***<br>*Residence*

SCHOOLING AND WORK OF THE CHILD

**A.4**	**School/Work (Column 6)**<br><br>*FILL IN THE YEARS THE CHILD STARTED AND FINISHED SCHOOL (INTERRUPT THE LINE WHEN THE CHILD WAS OUT OF SCHOOL FOR MORE THAN 6 MONTHS (EXPLAIN WHY IN A NOTE). COMPLETE Q.A.5 AND Q.A.6*<br>***IF THE CHILD IS NOT AT SCHOOL** AND IS WORKING, FILL IN THE WORKS OF THE CHILD THE SAME WAY **.***		***Column 6***<br>*School/ Work*
**A.5**	What kind of school does/did the child attend(ed)?	☐ 1 Public school<br>☐ 1 Private school<br>☐ 2 School at the temple<br>☐ 3 Other, specify:…………………..	
**A.6**	Did the child ever repeated a school year?	☐ 1 No<br>☐ 2 Yes, specify number of years:<br>…………………………………………………………	
**If the child is currently working**			
A.7.	What is his occupation?	…………………………………………………………	
A.8	Can you tell the child approximate income?	|__|__|,|__|__|__| Baht<br><br>☐ 1 Per month (last month)<br>☐ 2 Per day<br>If per day, specify how many days of work in the last month: |__|__|	
A.9	Does she child share his income with the household?	☐ 1 No<br>☐ 2 Yes	

SECTION C: HEALTH OF THE CHILD

Now I would like to ask you a few questions about the child's HIV infection history.

**C.1**	**Life Events Table: Disease (Column 7)** Can you tell me when/at what age the child was diagnosed as HIV infected<br>*FILL IN THE AGE OF THE CHILD IN COLUMN 7: **DISEASE*** **TEST**	|___|___|years or |___|___|month<br>☐ 98 Don't know	***Column 7*** *Disease*
C.2	What was the reason for the **diagnosis** to be made?<br>*Spontaneous comments:* ……………………………………………………<br>……………………………………………………<br>……………………………………………………<br>……………………………………………………<br>……………………………………………………<br>*Spontaneous comments on how the child was infected:*<br>☐ *Mother-to-child transmission:*<br>☐ *Other, specify:* ………………………………………	☐ 1 At the hospital because of HIV symptoms (specify):……………………………………<br>☐ 2 At the hospital for another reason (specify): ……………………………………………………<br>☐ 3 Because his/her parent was found HIV positive during pregnancy and the child was tested after birth<br>☐ 4 Because his/her parent was ill<br>☐ 5 Because his/her parent died of AIDS<br>☐ 6 Because of illness or death of a sibling<br>☐ 7 Other (specify): ……………………………… ……………………………………………………<br>☐ 98 Don't know	

HIV SYMPTOMS

**C.3**	**Life Events Table: Disease (Column 7)** Could you tell me at what age the child first experienced some signs/symptoms of HIV/AIDS?<br>*FILL IN THE AGE OF THE CHILD IN COLUMN 7: **DISEASE*** **SYMPT**	☐ 1 Never ➔ ***C.5***<br>|___|___|years or |___|___|month<br>☐ Don't know		***Column 7*** *Disease*
C.5	Did the child suffer any of the following disease<br>*Can be more than one answer*<br>*Comments:*……………………………………<br>……………………………………………………<br>……………………………………………………		Yes	No
		Tuberculosis	☐	☐
		Pneumonia	☐	☐
		Meningitis	☐	☐
		Parotitis	☐	☐
		Skin problem	☐	☐
		Growth retardation	☐	☐
		Mental retardation	☐	☐
		Other, specify:…………………	☐	☐
C.6	Does the child currently experience any symptoms or health problems?	☐ 1 No<br>☐ 2 Yes (specify): ……………………………<br>……………………………………………………<br>……………………………………………………		

ARV TREATMENT

**C.7**	**Life Events Table: Disease (Column 7)**<br>Could you tell me when/at what age the child first received ARV treatment?<br><br>***FILL IN THE AGE OF THE CHILD IN COLUMN 7: DISEASE*** **ARV**	|___|___|years old<br>☐ Don't know<br>*If the child did not initiate ARV treatment, Skip to* **C.14**	***Column 7***<br>*DISEASE*
C.8	Do you think that it easy or difficult for the child to remember to take the treatment?<br><br>*REFER TO THE "SMILE"* ***SCALE***	☐ 5 Very Easy<br>☐ 4 Easy<br>☐ 3 OK<br>☐ 2 Difficult<br>☐ 1 Very difficult	
C.9	Do you have to remind the child to take the treatment	☐ 1 Never ....<br>☐ 2 Sometimes<br>☐ 3 Often<br>☐ 4 Always because I am the person in charge of the treatment	➔ C11
C.10	When reminded to take the treatment, is the child compliant?	☐ 1 No<br>☐ 2 Yes<br>☐ 98 Don't know	
C.11	How do you evaluate the child's adherence to treatment<br><br>*REFER TO THE "SMILE"* ***SCALE***	☐ 5 Very Good<br>☐ 4 Good<br>☐ 3 Fair<br>☐ 2 Poor<br>☐ 1 Very poor	
C.12	Can you tell in which circumstances the child may forget to take the treatment? (the most frequent situation)<br>Only one answer<br>Comments: ....<br>...	☐ 0 Never forget<br>☐ 1 Sick<br>☐ 2 Too Busy<br>☐ 3 Not At Home<br>☐ 4 Festival/Holidays/Camps<br>☐ 5 With friends<br>☐ 6 Other (specify)	
C.14	Was the child hospitalized at least one night **for a health problem**?<br><br>*If yes and the reason is spontaneously mentioned, specify:*	☐ 1 Never **Skip to C.16**<br>☐ 2 Yes<br><br>..<br>..	
C.15	If yes, how many times?	Before ARV initiation: |__|__| After ARV initiation: |__|__| In the past 12 months |__|__| ☐ 98 Don't know	
C.16	**Life Events Table: Health Status (column 8)**<br>How would you rate the child health status throughout his life<br><br>*USE THE "****SMILE"*** ***SCALE***<br><br>*FILL IN THE HEALTH STATUS THROUGHOUT LIFE, IN PARTICULAR* ***BEFORE AND AFTER ARV***	**5**: VERY GOOD<br>**4**: GOOD<br>**3**: FAIR<br>**2**: POOR<br>**1**: VERY POOR	Life-event<br>**column 8**

HIV DISCLOSURE

C.17	Does the child know that he/she is infected with HIV?<br>*Spontaneous comments*:......................................<br>...<br>...	☐ 1 Yes<br>☐ 2 No<br>☐ 3 Not sure	
C.18	Was the child formally told about his HIV status?<br>*Spontaneous comments*:......................................<br>...<br>...	☐ 1 Yes<br>☐ 2 No, but knew by himself as he/she grew up ➔ D1<br>☐ 3 No ➔ D1	
**C.19**	**Life Events Table: Disease (Column *7*)**<br>Can you tell me when/at what age or the period the child knew about his/her HIV status	☐ 1 Never ➔ **D1**<br>At |___|___|years old<br>Or<br>between|___|___| and |___|___|years<br>☐ Don't know	***Column 7***<br>*Disease*
FILL IN THE AGE OF THE CHILD WHEN HE HAD SYMPTOMS IN COLUMN 7: DISEASE		**DISCLOS**	
C.20	Who told the child about his/her HIV status	....	|__|__|
C.21	If the child was told about his/her HIV status<br>Why was he/she told at that particular moment?<br>*Comments:* ....<br>...<br>...<br>...	☐ 1 Adequate age<br>☐ 2 Was asking about his/her disease/treatment<br>☐ 3 Heard it from friends or at school<br>☐ 4 Mom/Dad was ill/died<br>☐ 5 Child was ill<br>☐ 6 Other, specify:...................................<br>☐ 98 Don't know	
C.22	What was the child reaction at the time of disclosure?<br>*Comments*: ....<br>...<br>...<br>...	☐ 1 Relieved to know<br>☐ 2 Depressed<br>☐ 3 Angry<br>☐ 4 Non responsive<br>☐ 5 Other, specify:...................................<br>☐ 98 Don't know	
C.23	If the child was receiving ARV at that time, did the disclosure had an impact on his/her adherence<br>*Comments*: ....<br>...	☐ 1 No impact<br>☐ 2 Improved<br>☐ 3 Decreased<br>☐ 98 Don't know	

SECTION D: DISCRIMINATION

D.1	Do you think that people in your community know about the child's HIV status?<br>Comments: ....<br>...	☐ 1 Yes most of the community<br>☐ 2 Yes some of them<br>☐ 3 No<br>☐ 98 Don't know	
D.2	Do you think that the school staff knows about the child's HIV status?	☐ 1 Yes most of them<br>☐ 2 Yes some of them<br>☐ 3 No<br>☐ 98 Don't know	
D.3	Do you think that the students at school know about the child's HIV status?	☐ 1 Yes most students<br>☐ 2 Yes some students<br>☐ 3 No<br>☐ 98 Don't know	
D.4	**Household Form**<br>In your household, who knows about the child's HIV status?		**House-hold Form**
	FILL IN THE COLUMN D.3 OF THE HOUSEHOLD FORM		
D.5	Is there someone else who is HIV infected in the Household and is he/she receiving ARV?		

		Yes once	Yes regularly	No	Don't Know	NA
D.6	Do you know whether the child ever experienced stigma or discrimination related to his/her HIV status :					
	From family members	☐ 1	☐ 2	☐ 3	☐ 98	☐ 99
	From friends	☐ 1	☐ 2	☐ 3	☐ 98	☐ 99
	At school	☐ 1	☐ 2	☐ 3	☐ 98	☐ 99
	At work (if the child is working)	☐ 1	☐ 2	☐ 3	☐ 98	☐ 99
	At the hospital	☐ 1	☐ 2	☐ 3	☐ 98	☐ 99
	From people in the village /neighborhood	☐ 1	☐ 2	☐ 3	☐ 98	☐ 99
	Others:..	☐ 1	☐ 2	☐ 3	☐ 98	☐ 99

D.7	Is there a particular experience of stigmatization concerning the child that you can tell us about	....<br>..<br>..<br>..<br>..<br>..<br>..<br>..<br>..

SECTION E: HOUSEHOLD INCOME

E.1	Do you have a regular income from work?	☐ 1 No………………………………………<br>☐ 2 Yes	⇒QE.3
E.2	Please tell me your approximate income per month or per day from your work?	|__|__|,|__|__|__| Bahts<br><br>☐ 1 Per month (last month)<br>☐ 2 Per day<br>If per day, specify how many days of work in the last month: |__|__|	
E.3	**HOUSEHOLD INCOME** *You said that ____ persons where living in your house.*<br>Could you tell me the approximate income per month (or per year) of each household members?		**House-hold Form**
	FILL IN THE COLUMN* E.3 *OF THE HOUSEHOLD FORM		

		You	Partner	Child	Co-resident	
E.4	Do you receive support from the government on a regular basis?	☐ 1 Yes<br>☐ 2 No	☐ 1 Yes<br>☐ 2 No	☐ 1 Yes<br>☐ 2 No	☐ 1 Yes<br>☐ 2 No	
	Amount (Bahts)	|__|__|__|	|__|__|__|	|__|__|__|	|__|__|__|	
E.5	Do you or your household receive support from someone else or from local organizations on a regular basis?	☐ 1 Yes<br>☐ 2 No ………………………………………				⇒Q E.7
E.6	If yes, how much per month?	|__|__||__|__|__| Bahts				

E.7	Do you currently own the following items?	Don't have	Fully owned	Owned by relative	Mortgaged/ on loan
	House	☐ 0	☐ 1	☐ 2	☐ 3
	Land	☐ 0	☐ 1	☐ 2	☐ 3
	Car	☐ 0	☐ 1	☐ 2	☐ 3
	Motorcycle	☐ 0	☐ 1	☐ 2	☐ 3
	TV	☐ 0	☐ 1	☐ 2	☐ 3
	Refrigerator	☐ 0	☐ 1	☐ 2	☐ 3
	Mobile phone	☐ 0	☐ 1	☐ 2	☐ 3

E.8	What is your health coverage?<br><br>*Comments*: ....<br><br>..	☐ 0 None<br>☐ 1 Universal coverage<br>☐ 2 Social Security Scheme<br>☐ 3 Workmen Compensation Fund<br>☐ 4 Civil Servant Medical Benefit Scheme<br>☐ 5 Other (specify): ....
	What is the health coverage of your child?	☐ 0 None<br>☐ 1 Universal coverage<br>☐ 2 Social Security Scheme<br>☐ 3 Workmen Compensation Fund<br>☐ 4 Civil Servant Medical Benefit Scheme<br>☐ 5 Other (specify): ....
E.9	Do you pay the child ARV treatment?	☐ 1 Yes<br>☐ 2 No

	**SECTION F: LIFE STYLE**		
	This is the last section of the interview. We will just ask you a few questions about your lifestyle and how you perceive your child		
F.1	Are you a member of any groups that support people living with HIV or caregivers of children living with HIV?	☐ 1 Yes, specify……………………………… ☐ 2 No	
F.2	Do you follow a religion?	☐ 1 Yes ☐ 2 No ……………………………………	⇒**Q** F.4
F.3	If yes, what is your religion?	☐ 1 Buddhist ☐ 2 Christian ☐ 3 Muslim ☐ 4 Other (specify: ……………………)	
F.4	How would you qualify your current financial situation *REFER TO THE "SMILE"* ***SCALE*** Comments:……………………………………………………… ………………………………………………………………………	☐ 1 Very difficult ☐ 2 Difficult ☐ 3 Ok ☐ 4 good ☐ 5 Very good	
F.5	How would you qualify your current happiness *REFER TO THE "SMILE"* ***SCALE*** Comments:……………………………………………………… ………………………………………………………………………	☐ 1 Very unhappy ☐ 2 unhappy ☐ 3 Average ☐ 4 Happy ☐ 5 Very happy	
A.7	How would you grade the child's intellectual capacity? *REFER TO THE "SMILE"* ***SCALE*** Comments: …………………………………………………… ………………………………………………………………………	☐ 1 Very Low ☐ 2 Low ☐ 3 Average ☐ 4 Good ☐ 5 Very good	
F.6	Do you think that the child is *REFER TO THE "SMILE"* ***SCALE*** Comments:……………………………………………………… ………………………………………………………………………	☐ 1 Very unhappy ☐ 2 Unhappy ☐ 3 Average ☐ 4 Happy ☐ 5 Very happy	

F.7	Do you think that the child is<br><br>*REFER TO THE "SMILE"* ***SCALE***<br><br>Comments:…………………………………………………<br><br>……………………………………………………………	☐ 1 Very disobedient<br>☐ 2 Somehow disobedient<br>☐ 3 Average<br>☐ 4 Nice<br>☐ 5 Very nice	
F.9	Can you evaluate your communication with the child<br><br>*REFER TO THE "SMILE"* ***SCALE***<br><br>Comments:…………………………………………………<br><br>……………………………………………………………	☐ 1 Very difficult<br>☐ 2 Difficult<br>☐ 3 OK<br>☐ 4 Easy<br>☐ 5 Very easy	
F.8	Does the child help at home<br><br>*More than one anwer possible*<br><br>Comments:…………………………………………………<br><br>……………………………………………………………	☐ 0 No	☐ 0 Yes,*specify*<br>☐ 1 cleaning the house?<br>☐ 2 shopping<br>☐ 3 cooking<br>☐ 4 looking after younger relatives<br>☐ 5 cleaning the dishes<br>☐ 6 doing the laundry/ironing<br>☐ 7 helping with parents activities (shop, farm, etc.)<br>☐ 8 Other (specify):
F.10	Do you have conflicts with the child?<br><br>Comments:…………………………………………………<br><br>……………………………………………………………	☐ 1 All the times<br>☐ 2 sometimes<br>☐ 3 rarely<br>☐ 4 Never	
F.11	In case of conflict, what is the main reason for the conflicts?: ……………………… ….<br>…………………………………………………………………………………… ….		

F.13	Do you have any comments about the questionnaire: ……………………………… ….<br>…………………………………………………………………………………… ….<br>…………………………………………………………………………………… ….	

Thank you very much for your cooperation. Even if you felt some questions were a bit personal, you should be aware that your answers are important in order to better understand the situation of HIV infected adolescents in Thailand and to define programs to improve the situation at the national level.

SECTION I. INTERVIEWER'S COMMENTS

	Time interview ended:|__|__| Hour |__|__| Minutes		
I.1	The interview was performed	☐ 1 Without any interruption<br>☐ 2 The person showed signs that he/she wanted to stop the interview<br>☐ 3 Was interrupted several times	
I.2	Describe the person interviewed :<br><br>*(Can be more than one answer)*	☐ 1 Was relaxed<br>☐ 2 Was OK<br>☐ 3 Was very emotional<br>☐ 4 Was watching at the time<br>☐ 5 Had difficulty understanding<br>☐ 6 Had difficulty expressing her/himself<br>☐ 7 Had difficulty remembering or dating events<br>☐ 8 Was physically tired	
I.3	The answers seemed	☐ 1 Very reliable<br>☐ 2 Reliable<br>☐ 3 Not reliable<br>☐ 4 Not at all reliable<br>☐ 98 Don't know	
