# Supplementary material for: Factors influencing antiretroviral treatment suboptimal adherence among perinatally HIV-infected adolescents in Thailand
Source: PLoS One. 2017 Feb 16;12(2):e0172392. doi: 10.1371/journal.pone.0172392 (PMC5312953; doi:10.1371/journal.pone.0172392)
Supplement: S4 File — (PDF) [file pone.0172392.s004.pdf]

สถาบันการศึกษาประชากรแห่งชาติ ปารีส
สถาบันวิจัยสังคม มหาวิทยาลัยเชียงใหม่ จังหวัดเชียงใหม่

แบบสอบถามสำหรับพ่อแม่/ผู้ดูแล

เมษายน 2553

รหัสประจำตัวผู้ให้สัมภาษณ์ : [ ] หมายเลขประจำตัวเด็ก: ....

เด็ก

เพศของเด็กในความดูแล: ☐ 1 ชาย ☐ 2 หญิง

วันที่สัมภาษณ์: |__|__| วัน |__|__| เดือน|_2_|_5_|_5_|_ _| ปี

เวลาเริ่มสัมภาษณ์: |__|__| นาฬิกา |__|__| นาที

กรุณาแนะนำโครงการวิจัยและขอการแสดงความยินยอม

ชื่อผู้สัมภาษณ์: .... รหัส: |__|__|

สถานที่ที่เข้าร่วมการวิจัย: .... รหัส: |__|__|

หมวด R: ผู้ตอบแบบสอบถาม

เราต้องการทราบเกี่ยวกับท่านในฐานะที่ท่านเป็นผู้ดูแลเด็ก			
R.0	เพศ	☐ 1 ชาย<br>☐ 2 หญิง	
R.00	อายุของผู้ตอบแบบสอบถาม	|__|__| ปี	
R.1	ท่านมีความสัมพันธ์กับเด็กอย่างไร	....	|__|__|
R.2	สถานภาพสมรสของท่านคือ	☐ 1 โสด<br>☐ 2 สมรส<br>☐ 3 หย่า/ แยกกันอยู่<br>☐ 4 หม้าย	
G.1	สัญชาติของท่านคือ?	☐ 1 สัญชาติไทย<br>☐ 2 ไม่ใช่สัญชาติไทย ระบุ.............................	
G.2	เชื้อชาติของท่านคือ?	☐ 1 เชื้อชาติไทย<br>☐ 2 ไม่ใช่เชื้อชาติไทย ระบุ.............................	
R.3	ท่านเคยเข้าโรงเรียนหรือไม่	☐ 1 ใช่<br>☐ 2 ไม่	⇒**Q** R.5
R.4	ท่านสำเร็จการศึกษาในระดับใด	ระดับการศึกษาสูงสุด: |__|__| ☐ 1 ประถมศึกษา<br>☐ 2 มัธยมศึกษาตอนต้น<br>☐ 3 วิทยาลัยครู<br>☐ 4 วิทยาลัยอาชีวศึกษา<br>☐ 5 มหาวิทยาลัย<br>☐ 6 อื่นๆ (ระบุ)..	
R.5	ปัจจุบันท่านประกอบอาชีพอะไร	....	|__|__|
R.6	ท่านจัดลำดับคะแนนด้านสุขภาพของท่านอยู่ในระดับไหน ใช้ "*SMILE" **SCALE***	☐ 1 แย่มาก<br>☐ 2 แย่<br>☐ 3 พอใช้<br>☐ 4 ดี<br>☐ 5 ดีมาก	|__|__|
R.7	ท่านป่วยเป็นโรคเรื้อรังใด(รวมถึงการติดเชื้อเอช ไอ วี) หรือทุพพลภาพถาวรหรือไม่	☐ 1 ไม่<br>☐ 2 ใช่ ระบุ	

หมวด A : คำถามเกี่ยวกับชีวิตของเด็ก

ลำดับ	คำถามหลักและคำถามสนับสนุน	ประเภทของการกรอกรหัส	ไปยัง
	**เด็ก :**		
	ลำดับแรก เราจะขอถามท่านเป็นคำถามเกี่ยวกับเด็กที่ท่านดูแล		
**A.1.**	**อายุ (column 2)**<br>เด็กอายุเท่าไร?<br>*วงกลมรอบอายุของเด็ก*	|___|___|ปี จนถึงวันนี้	***Column 2***<br>อายุ
**A.2**	**ปี (Column 1)**<br>เด็กเกิดวันที่เท่าไร (ปีที่เด็กเกิด)?	|___|___| |___|___| |_2_|_5_|___|___| วัน เดือน ปี	***Column 1***<br>ปี
	1. *กรอกปีที่เกิด และทุกๆปีต่อมาจนกระทั่งถึงปีปัจจุบัน*<br>*2. ขีดเส้นสำหรับปีปัจจุบัน*		

G. 3	สัญชาติของเด็กคือ?	☐ 1 สัญชาติไทย<br>☐ 2 ไม่ใช่สัญชาติไทย ระบุ..............................	
G. 4	เชื้อชาติของเด็กคือ?	☐ 1 เชื้อชาติไทย<br>☐ 2 ไม่ใช่เชื้อชาติไทย ระบุ..............................	|__|__|

สถานภาพของบิดามารดาผู้ให้กำเนิดเด็ก

	**มารดา**		
B.3	***ถ้าผู้ตอบแบบสอบถามไม่ใช่มารดาของเด็ก ให้ถามคำถามดังต่อไปนี้***<br>มารดาของเด็กยังมีชีวิตอยู่หรือไม่:	☐ 1 ใช่<br>☐ 2 อาศัยอยู่ในครอบครัว ➔ ***B.6***<br>☐ 3 ไม่ได้อาศัยอยู่ในครอบครัว ➔ ***B.6***<br>**ท่านต้องกรอกแบบสอบถามในหัวข้อ *BB* ในหน้าถัดไป**<br>☐ 4 ไม่ ☐ 98 ไม่ทราบ	
B.4	*กรอกวันเดือนปีในคอลัมน์สถานภาพของมารดา (COLUMN 3) และ**อายุของเด็กขณะที่มารดาของเด็กเสียชีวิต*** ***MDTH***		***Column 3***
B.5	กรณีที่มารดาของเด็กเสียชีวิต การเสียชีวิตนั้นเกี่ยวข้องกับ เอช ไอ วี / เอดส์ หรือไม่	☐ 1 ใช่ ระบุ...<br>☐ 2 ไม่ใช่ ระบุ:...<br>☐ 98 ไม่ทราบ	
	**บิดา**		
B.6	***ถ้าผู้ตอบแบบสอบถามไม่ใช่บิดาของเด็ก ให้ถามคำถามดังต่อไปนี้***<br>บิดาของเด็กยังมีชีวิตอยู่หรือไม่:	☐ 1 ใช่<br>☐ 2 อาศัยอยู่ในครอบครัว ➔ ***B.9***<br>☐ 3 ไม่ได้อาศัยอยู่ในครอบครัว ➔ ***B.9***<br>**ท่านต้องกรอกแบบสอบถามในหัวข้อ *BB* ในหน้าถัดไป**<br>☐ 4 ไม่ ☐ 98 ไม่ทราบ	
B.7	*กรอกวันเดือนปีในคอลัมน์สถานภาพของบิดา (COLUMN 3) และ**อายุของเด็กขณะที่บิดาของเด็กเสียชีวิต** **FDTH***		***Column3***
B.8a	กรณีที่บิดาของเด็กเสียชีวิต การเสียชีวิตนั้นเกี่ยวข้องกับ เอช ไอ วี / เอดส์ หรือไม่	☐ 1 ใช่ ระบุ...<br>☐ 2 ไม่ใช่ ระบุ:...<br>☐ 98 ไม่ทราบ	
B.8b	ถ้าพ่อแม่ของเด็กหย่าร้าง ให้เขียนลงใน MF-SEP ปีที่หย่าร้าง<br>M F-SEP|__|__|ปี หรืออายุของเด็ก |__|__|		***Column3***
	**พี่น้องของเด็ก (พ่อแม่เดียวกันเท่านั้น)**		
B.9	เด็กมีพี่น้องกี่คน รวมพี่น้องที่เสียชีวิตไปแล้ว<br>กรณีที่มีการกล่าวถึงพี่น้องคนอื่นๆ (ที่มิได้ถือกำเนิดจากบิดามารดาเดียวกัน) ระบุ..........................<br>..	|__|__| รวมทั้งตัวเด็กด้วย	
B.10	พี่น้องที่ยังมีชีวิตอยู่ มีกี่คน	|__|__| ☐ 98 ไม่ทราบ	
B.11	มีพี่น้องกี่คนที่ติดเชื้อเอช ไอ วี	|__|__| ☐ 98 ไม่ทราบ	
B.12	มีพี่น้องกี่คนที่เสียชีวิตเนื่องจากเอช ไอ วี /	|__|__| ☐ 98 ไม่ทราบ	

ผู้ดูแลเด็ก

B.13	ตารางเหตุการณ์ในชีวิต: ผู้ดูแลเด็ก **(Column 4)**<br>*ระบุความสัมพันธ์ของผู้ดูแลกับเด็กตั้งแต่แรกคลอด (ความสัมพันธ์ในฐานะผู้ดูแลที่แท้จริง)*	***Column 4***<br>ผู้ดูแล
	ต่อไปนี้จะขอถามเกี่ยวกับผู้ที่ร่วมอาศัยกับเด็ก	

เราต้องการทราบเกี่ยวกับผู้ที่ร่วมอาศัยกับเด็ก

	**แบบสอบถามด้านครัวเรือน**	**ผู้ร่วมอาศัย**	**แบบสอบถามด้านครัวเรือน**
H.1	1. กรอกข้อมูลในส่วน **H** ของแบบสอบถามด้านครัวเรือน;<br>2. ให้ข้อมูลของผู้ร่วมอาศัยเป็นรายบุคคล กรอกอายุของแต่ละคน **ความสัมพันธ์ของผู้ที่ร่วมอาศัยกับเด็ก** อาชีพของผู้ร่วมอาศัย		

หัวข้อ BB คำถามในส่วนนี้สำหรับกรณีที่มารดายังมีชีวิตอยู่แต่ไม่ได้อาศัยอยู่กับครอบครัวหรือบิดายังมีชีวิตอยู่แต่ไม่ได้อาศัยอยู่กับครอบครัวเท่านั้น

	**สถานภาพของบิดา**	**สถานภาพของมารดา**
BB.1	บิดาของเด็กติดเชื้อเอช ไอ วีหรือไม่<br>☐ 1 ใช่<br>☐ 2 ไม่<br>☐ 98 ไม่ทราบ	มารดาของเด็กติดเชื้อเอช ไอ วีหรือไม่<br>☐ 1 ใช่<br>☐ 2 ไม่<br>☐ 98 ไม่ทราบ
BB.2	บิดาของเด็กอาศัยอยู่ที่ไหน...........................	มารดาของเด็กอาศัยอยู่ที่ไหน..................................
BB.3	บิดาของเด็กประกอบอาชีพอะไร..........................	มารดาของเด็กประกอบอาชีพอะไร.............................
BB.4	บิดาของเด็กได้ส่งเงินมาให้เพื่อใช้เลี้ยงดูเด็กหรือไม่<br>☐ 1 ใช่<br>☐ 2 ไม่	มารดาของเด็กได้ส่งเงินมาให้เพื่อใช้เลี้ยงดูเด็กหรือไม่<br>☐ 1 ใช่<br>☐ 2 ไม่
BB.5	สถานการณ์ของครอบครัวบิดาเป็นอย่างไร<br>☐ 1 ยังคงสถานภาพสมรส<br>☐ 2 หย่าร้าง<br>☐ 3 แต่งงานใหม่<br>☐ 4 แต่งงานใหม่และมีลูกติดจากภรรยาใหม่<br>☐ 5 อื่นๆ..<br>☐ 98 ไม่ทราบ	สถานการณ์ของครอบครัวมารดาเป็นอย่างไร<br>☐ 1 ยังคงสถานภาพสมรส<br>☐ 2 หย่าร้าง<br>☐ 3 แต่งงานใหม่<br>☐ 4 แต่งงานใหม่และมีลูกติดจากสามีใหม่<br>☐ 5 อื่นๆ..<br>☐ 98 ไม่ทราบ
BB.6	เด็กได้พบกับบิดาบ่อยแค่ไหน**<u>ในปีที่ผ่านมา</u>**<br>☐ 1 มากกว่า 1 ครั้งต่อสัปดาห์<br>☐ 2 เดือนละ 2 – 3 ครั้ง<br>☐ 3 ปีละ 2 – 3 ครั้ง<br>☐ 4 ปีละครั้ง<br>☐ 5 ไม่เคยพบ<br>☐ 6 อื่นๆ (ระบุ):<br>(ตอบข้อเดียวที่เป็นไปได้เท่านั้น)	เด็กได้พบกับมารดาบ่อยแค่ไหน**<u>ในปีที่ผ่านมา</u>**<br>☐ 1 มากกว่า 1 ครั้งต่อสัปดาห์<br>☐ 2 เดือนละ 2 – 3 ครั้ง<br>☐ 3 ปีละ 2 – 3 ครั้ง<br>☐ 4 ปีละครั้ง<br>☐ 5 ไม่เคยพบ<br>☐ 6 อื่นๆ (ระบุ):<br>(ตอบข้อเดียวที่เป็นไปได้เท่านั้น)

ที่อยู่อาศัยของเด็ก

	ต่อไปนี้จะขอถามคุณ**เกี่ยวกับสถานที่ที่เด็ก**อยู่อาศัยมานานกว่า 1 ปีตลอดช่วงชีวิตของเด็ก	
A.3	ที่อยู่อาศัย **(Column 5)**<br>พักอยู่ที่ไหน (ตัวเมือง/หมู่บ้าน) ที่เด็กพักอาศัยอยู่ตั้งแต่เกิดและเริ่มพักอาศัยเมื่อใด<br>*กรอกชื่อที่พักทั้งหมดที่เด็กเคยพักอาศัยอยู่ (ตัวเมือง/หมู่บ้าน)* ***เป็นเวลาอย่างน้อย 1 ปีตั้งแต่เกิดจนถึงปีปัจจุบัน***	***Column 5***<br>ที่อยู่อาศัย

การไปโรงเรียนและการทำงานของเด็ก

**A.4**	*การเข้าชั้นเรียน / การทำงาน* ***(Column 6)***<br>*กรอกปีที่เด็กเริ่มเข้าเรียนและเรียนจบ เว้นระยะบรรทัดในช่วงที่เด็กหยุดเรียนมากกว่า 6 เดือน(อธิบายว่าเพราะเหตุใด)*<br>*กรอกแบบสอบถามในข้อ QA5และQA6*<br>***ถ้าเด็กไม่ได้ไปโรงเรียนและกำลังทำงานอยู่ให้กรอกงานที่เด็กทำ****ในลักษณะเดียวกัน*		***Column 6***<br>โรงเรียน/<br>ที่ทำงาน
**A.5**	เด็กเข้าเรียน/เคยเรียนในโรงเรียนประเภทไหน	☐ 1 โรงเรียนรัฐบาล<br>☐ 2 โรงเรียนเอกชน<br>☐ 3 โรงเรียนวัด<br>☐ 4 อื่นๆ ระบุ:...	
**A.6**	เด็กเคยเรียนซ้ำชั้นหรือไม่	☐ 1 ไม่<br>☐ 2 ใช่ ระบุระยะเวลา (กี่ปี):<br>..	
**เด็กกำลังทำงานอยู่หรือไม่?**			
A.7.	เด็กประกอบอาชีพอะไร?	....	
A.8	ท่านสามารถบอกรายได้โดยประมาณของเด็กได้หรือไม่?	|__|__|,|__|__|__| บาท<br>☐ 1 ต่อเดือน (*เดือนที่ผ่านมา*)<br>☐ 2 ต่อวัน<br>*ถ้ามีรายได้ต่อวัน*<br>*ระบุว่าทำงานกี่วันในเดือนในเดือนที่ผ่านมา*: |__|__|	
A.9	เด็กแบ่งเงินรายได้ให้ครอบครัวหรือไม่?	☐ 1 ไม่<br>☐ 2 ใช่	

หมวด C: สุขภาพของเด็ก

ต่อไปนี้จะขอถาม 2 – 3 คำถามเกี่ยวกับประวัติการติดเชื้อเอช ไอ วีของเด็ก

**C.1**	**ตารางเหตุการณ์ในชีวิต: โรค (Column 7)**<br>ท่านช่วยบอกว่าเด็กได้รับการวินิจฉัยว่าติดเชื้อเอช ไอ วี เมื่ออายุเท่าไหร่<br>กรอกอายุเด็กในคอลัมน์ 7 **;** โรค	|___|___|ปี หรือ |___|___| เดือน<br>☐ 98 ไม่ทราบ<br>***TEST***	***Column 7***<br>โรค
C.2	เหตุผลที่เด็กได้รับ**การวินิจฉัย**คืออะไร<br>*ข้อคิดเห็นที่ตอบตามธรรมชาติ*:<br>...<br>...<br>...<br>...<br>อธิบายตามธรรมชาติว่าเด็กติดเชื้อได้อย่างไร *:*<br>☐ *การถ่ายทอดเชื้อจากแม่สู่ลูก*<br>☐ *อื่นๆ ระบุ: ....*	☐ 1 เนื่องจากเกิดอาการแสดงของการติดเชื้อ เอช ไอ วี (ระบุ):..<br>☐ 2 ที่โรงพยาบาลเนื่องจากเหตุผลอื่นๆ (ระบุ): ....<br>☐ 3 เนื่องจากบิดา/มารดาของเด็กติดเชื้อเอช ไอ วีขณะตั้งครรภ์ และเด็กได้รับการตรวจเพื่อหาการติดเชื้อหลังคลอด<br>☐ 4 เนื่องจากบิดามารดาของเด็กป่วย<br>☐ 5 เนื่องจากบิดามารดาของเด็กเสียชีวิตเนื่องจากโรคเอดส์<br>☐ 6 เนื่องจากพี่น้องเจ็บป่วยหรือเสียชีวิต<br>☐ 7 อื่นๆ (ระบุ):...<br>...<br>☐ 98 ไม่ทราบ	

อาการแสดงของเอช ไอ วี

**C.3**	**ตารางเหตุการณ์ในชีวิต:โรค (Column 7)**<br>ท่านช่วยบอกว่าเด็กมีอาการ/อาการแสดงต่างๆอันเนื่องมาจากการติดเชื้อเอช ไอ วี / เอดส์เมื่อไหร่<br>กรอกอายุเด็กในคอลัมน์ 7 **;** โรค **SYMPT**	☐ 0 ไม่เคย ➔ ***C.5***<br>|___|___|ปี หรือ |___|___| เดือน<br>☐ ไม่ทราบ		***Column 7***<br>โรค
C.4	ถ้าเคย เด็กต้องทนทุกข์ทรมานกับโรคต่อไปนี้หรือไม่<br>ตอบได้มากกว่า **1** ข้อ<br>ข้อเสนอแนะ:..<br>...<br>...		ใช่	ไม่
		วัณโรค	.............☐	☐
		ปอดอักเสบ	.............☐	☐
		เชื้อราที่เยื่อหุ้มสมอง	.............☐	☐
		คางทูม	.............☐	☐
		ปัญหาโรคผิวหนัง	.............☐	☐
		การเจริญเติบโตล่าช้า	.............☐	☐
		ความบกพร่องทางสติปัญญา	.............☐	☐
		อื่นๆ ระบุ:...............	.............☐	☐
C.5	ปัจจุบันนี้เด็กมีอาการแสดงต่างๆของโรคหรือมีปัญหาด้านสุขภาพหรือไม่	☐ 1 ไม่<br>☐ 2 ใช่ (ระบุ):..		

การรักษาด้วยยาต้านไวรัส

C.6	**ตารางเหตุการณ์ในชีวิต:โรค (Column 7)**<br>ท่านช่วยบอกว่าเด็กได้รับการรักษาด้วยยาต้านไวรัสครั้งแรกเมื่อไหร่<br><br>กรอกอายุเด็กในคอลัมน์ 7 **:** โรค **ARV**	|___|___|ปี<br>☐99 ไม่ทราบ<br>*ถ้าเด็กยังไม่ได้เริ่มรับรับการรักษาด้วยยาต้านไวรัส ข้ามไปข้อ* **C.14**	***Column 7***<br>โรค
C.7	ท่านคิดว่าเป็นเรื่องง่ายหรือยากสำหรับเด็กในการจดจำว่าต้องรับประทานยา<br>*ใช้* ***"SMILE" SCALE***	☐5 ง่ายมาก<br>☐4 ง่าย<br>☐3 ธรรมดา<br>☐2 ยาก<br>☐1 ยากมาก	
C.8	ท่านต้องคอยเตือนเด็กให้รับประทานยาหรือไม่	☐1 ไม่เคย ....<br>☐2 บางครั้ง<br>☐3 บ่อยๆ<br>☐4 เป็นประจำเนื่องจากผม/ดิฉันเป็นผู้ที่รับผิดชอบเรื่องการรับประทานยาของเด็ก	➔ C11
C.9	เวลาที่เตือนให้เด็กรับประทานยา เด็กปฏิบัติตามหรือไม่	☐1 ไม่<br>☐2 ใช่<br>☐98 ไม่ทราบ	
C.10	ท่านประเมินความร่วมมือในการรับประทานยาของเด็กเป็นอย่างไร<br><br>*ใช้* ***"SMILE" SCALE***	☐5 ดีมาก<br>☐4 ดี<br>☐3 พอใช้<br>☐2 แย่<br>☐1 แย่มาก	
C.11	ท่านช่วยบอกว่าสถานการณ์แบบไหนที่เด็กอาจลืมรับประทานยามากที่สุด<br><br>ข้อเสนอแนะ:	☐0 ไม่เคยลืม<br>☐1 ป่วย<br>☐2 ยุ่งมากๆ<br>☐3 ไม่ได้อยู่บ้าน<br>☐4 ช่วงเทศกาล/วันหยุด/ไปเข้าค่าย<br>☐5 อยู่กับเพื่อน<br>☐6 อื่นๆ (ระบุ)	
C.12	เด็กเคยเข้ารับการรักษาในโรงพยาบาลเป็นเวลาอย่างน้อย 1 คืนอันเนื่องจากปัญหาสุขภาพ หรือไม่<br>ถ้าเคยเหตุผลที่กล่าวถึงอย่างเป็นธรรมชาติ ระบุ:	☐1 ไม่ **ข้ามไปข้อ C.16**<br>☐2 ใช่<br>..	
C.13	ถ้าเคย กี่ครั้ง	ก่อนเริ่มรับการรักษาด้วยยาต้านไวรัส**:** |__|__| หลังจากรับการรักษาด้วยยาต้านไวรัส**:** |__|__| ในช่วง 12 เดือนที่ผ่านมา |__|__| ☐98 ไม่ทราบ	
C.14	**ตารางเหตุการณ์ในชีวิต: สถานภาพทางด้านสุขภาพ (column 8)**<br>ท่านจัดลำดับสถานภาพทางด้านสุขภาพตลอดช่วงชีวิตของเด็กอย่างไร ใช้ ***"SMILE" SCALE***<br>กรอกข้อมูลสถานภาพทางด้านสุขภาพตลอดช่วงชีวิตของเด็กแบบเจาะจง **ก่อนและหลังรับประทานยาต้านไวรัส**	☐5 ดีมาก<br>☐4 ดี<br>☐3 พอใช้<br>☐2 แย่<br>☐1 แย่มาก	**เหตุการณ์ในชีวิต column 8**

การเปิดเผยสถานะการติดเชื้อเอช ไอ วี

C.1 5	เด็กทราบหรือไม่ว่าตัวเองติดเชื้อเอช ไอ วี<br>ข้อคิดเห็นที่ตอบตามธรรมชาติ:...............................<br>...<br>...	☐ 1 ทราบ<br>☐ 2 ไม่ทราบ<br>☐ 3 ไม่แน่ใจ	
C.1 6	เด็กได้รับการบอกสถานะการติดเชื้อเอช ไอ วี ของตนเองหรือไม่<br>ข้อคิดเห็นที่ตอบตามธรรมชาติ:...............................<br>..<br>...	☐ 1 ใช่<br>☐ 2 ไม่ แต่เด็กทราบเองเมื่อเด็กโตขึ้น ➔ **D1**<br>☐ 3 ไม่ ➔ **D1**	
**C.1 7**	**ตารางเหตุการณ์ในชีวิต: โรค (Column 7)**<br>ท่านช่วยบอกว่าเด็กทราบสถานะการติดเชื้อของตนเองเมื่อเด็กอายุเท่าไหร่หรือเมื่อช่วงอายุเท่าไหร่	☐ 0 **ไม่เคย ➔ D1**<br>เมื่อ |___|___|ปี<br>หรือ<br>ระหว่าง |___|___| และ |___|___|ปี<br>☐ **ไม่ทราบ**	***Column 7*** *โรค*
	**กรอกอายุของเด็กเมื่อเด็กมีอาการแสดงของการติดเชื้อเอช ไอ วี ในคอลัมน์ที่ *7: โรค***	***DISCLOSE***	
C.1 8	ใครบอกเด็กเกี่ยวกับสถานะการติดเชื้อเอช ไอ วี	....	|__|__|
C. 19	หากเด็กได้รับการบอกกกล่าวอย่างเป็นทางการแล้ว เพราะเหตุใดจึงบอกเด็ก ณ.ขณะนั้น<br>ข้อคิดเห็น: ....<br>...<br>...<br>...	☐ 1 อายุเหมาะสม<br>☐ 2 ถูกถามเกี่ยวกับโรค/การรักษาที่เด็กได้รับ<br>☐ 3 ได้ยินมาจากเพื่อนหรือจากที่โรงเรียน<br>☐ 4 มารดา/บิดา เจ็บป่วย/เสียชีวิต<br>☐ 5 เด็กป่วย<br>☐ 6 อื่นๆ ระบุ:...<br>☐ 98 ไม่ทราบ	
C. 20	ปฏิกิริยาของเด็ก ในขณะที่บอกให้ทราบเกี่ยวกับเรื่องนี้เป็นอย่างไร<br>ข้อคิดเห็น:<br>...<br>...	☐ 1 ผ่อนคลาย<br>☐ 2 หดหู่ หมดกำลังใจ<br>☐ 3 โกรธ<br>☐ 4 ไม่ตอบสนองใดๆ<br>☐ 5 อื่นๆ ระบุ:...<br>☐ 98 ไม่ทราบ	
C. 21	ถ้าเด็กกำลังรับการรักษาด้วยยาต้านไวรัสในขณะนั้น การเปิดเผยสถานภาพการติดเชื้อ มีผลกระทบต่อความร่วมมือในการรักษาหรือไม่<br>ข้อคิดเห็น:..<br>...	☐ 1 ไม่มีผลกระทบ<br>☐ 2 ความร่วมมือในการรักษาเพิ่มขึ้น<br>☐ 3 ความร่วมมือในการักษาลดลง<br>☐ 4 ไม่ทราบ	

หมวด D: การแบ่งแยกทางสังคม

D.1	ท่านคิดว่าคนในชุมชนทราบเกี่ยวกับสถานะการติดเชื้อเอช ไอ วีของเด็กหรือไม่<br>ข้อเสนอแนะ:	☐ 1 ใช่ คนในชุมชนส่วนใหญ่ทราบ<br>☐ 2 ใช่ ทราบเป็นบางคน<br>☐ 3 ไม่ทราบ<br>☐ 98 ไม่รู้	
D.2	ท่านคิดว่าครูที่โรงเรียนทราบเกี่ยวกับสถานะการติดเชื้อเอช ไอ วีของเด็กหรือไม่	☐ 1 ใช่ คนส่วนใหญ่ทราบ<br>☐ 2 ใช่ ทราบเป็นบางคน<br>☐ 3 ไม่ทราบ<br>☐ 98 ไม่รู้	
D.3	ท่านคิดว่านักเรียนที่โรงเรียนทราบเกี่ยวกับสถานะการติดเชื้อเอช ไอ วี ของเด็กหรือไม่	☐ 1 ใช่ นักเรียนส่วนใหญ่ทราบ<br>☐ 2 ใช่ ทราบเป็นบางคน<br>☐ 3 ไม่ทราบ<br>☐ 98 ไม่รู้	
D.4	**แบบฟอร์มด้านครัวเรือน**<br>ในครอบครัวของท่าน มีใครทราบสถานะการติดเชื้อเอช ไอ วี ของเด็ก		**แบบฟอร์มด้านครัวเรือน**
	กรอกข้อมูลลงในคอลัมน์ D.3 ของแบบสำรวจด้านครัวเรือน		
D.5	มีใครในครอบครัวของท่านที่ติดเชื้อเอช ไอ วีและเขา / เธอ กำลังรับประทานยาต้านไวรัสอยู่หรือไม่		

	ท่านทราบหรือไม่ว่าเด็กเคยประสบกับการถูกตีตราหรือการถูกแบ่งแยกทางสังคมที่สัมพันธ์กับสถานะการติดเชื้อเอช ไอ วีของเด็ก:	ใช่ หนึ่งครั้ง	ใช่บ่อยครั้ง	ไม่	ไม่ทราบ	**NA**
D.6	จากสมาชิกในครอบครัว	☐ 1	☐ 2	☐ 3	☐ 98	☐ 99
	จากเพื่อน	☐ 1	☐ 2	☐ 3	☐ 98	☐ 99
	ที่โรงเรียน	☐ 1	☐ 2	☐ 3	☐ 98	☐ 99
	ที่ทำงาน (ถ้าเด็กทำงาน)	☐ 1	☐ 2	☐ 3	☐ 98	☐ 99
	ที่โรงพยาบาล	☐ 1	☐ 2	☐ 3	☐ 98	☐ 99
	จากคนในหมู่บ้าน /เพื่อนบ้าน	☐ 1	☐ 2	☐ 3	☐ 98	☐ 99
	อื่นๆ:...	☐ 1	☐ 2	☐ 3	☐ 98	☐ 99

D.7	คุณช่วยบอกเล่าเกี่ยวกับประสบการณ์การถูกตีตราของเด็ก	....<br>..<br>..<br>..<br>..<br>..<br>..

หมวด E: รายได้ครัวเรือน

E.1	ท่านมีรายได้ประจำจากการทำงานหรือไม่	☐ 1 ไม่<br>☐ 2 ใช่	⇒QE.3
E.2	รายได้ของท่านโดยประมาณต่อเดือนหรือต่อวัน	|__|__|,|__|__|__| บาท<br>☐ 1 ต่อเดือน (*เดือนที่ผ่านมา*)<br>☐ 2 ต่อวัน<br>*ถ้ามีรายได้ต่อวัน*<br>*ระบุว่าทำงานกี่วันในเดือนในเดือนที่ผ่านมา*: |__|__|	
E.3	**รายได้ครัวเรือน**<br>ท่านบอกว่ามีสมาชิกจำนวน _____ คน ที่อาศัยอยู่ในบ้านของท่าน<br>ท่านช่วยบอกรายได้ครัวเรือนโดยประมาณต่อเดือนหรือต่อปีของแต่ละคนได้หรือไม่		**แบบสำรวจด้านครัวเรือน**
	กรอกข้อมูลในคอลัมน์ E.3 แบบสำรวจด้านครัวเรือน		

		ท่าน	คู่ครอง	เด็ก	ผู้ร่วมอาศัย
E.4	โดยปกติ<br>ท่านได้รับเงินสนับสนุนจากรัฐบาลเป็นประจำสม่ำเสมอหรือไม่	☐ 1 ใช่<br>☐ 2 ไม่	☐ 1 ใช่<br>☐ 2 ไม่	☐ 1 ใช่<br>☐ 2 ไม่	☐ 1 ใช่<br>☐ 2 ไม่
		จำนวน (บาท)			
		|__|__|__|	|__|__|__|	|__|__|__|	|__|__|__|

E.5	ท่านหรือครอบครัวของท่านได้รับการช่วยเหลือสนับสนุนจากบุคคลอื่นหรือจากองค์กรส่วนท้องถิ่นใดเป็นประจำสม่ำเสมอหรือไม่	☐ 1 ใช่<br>☐ 2 ไม่	⇒QE. 7
E.6	ถ้าใช่ ท่านได้รับจำนวนเท่าไหร่ต่อเดือน	|__|__||__|__|__| บาท	

	ปัจจุบันคุณมีสิ่งของตามรายการข้างล่างนี้เป็นของตัวเองหรือไม่	ไม่มี	เป็นของตนเอง	เป็นของญาติ	จำนอง/กู้ยืม
E.7	บ้าน	☐ 0	☐ 1	☐ 2	☐ 3
	ที่ดิน	☐ 0	☐ 1	☐ 2	☐ 3
	รถยนต์	☐ 0	☐ 1	☐ 2	☐ 3
	จักรยานยนต์	☐ 0	☐ 1	☐ 2	☐ 3
	โทรทัศน์	☐ 0	☐ 1	☐ 2	☐ 3
	ตู้เย็น	☐ 0	☐ 1	☐ 2	☐ 3
	โทรศัพท์มือถือ	☐ 0	☐ 1	☐ 2	☐ 3

E.8a	หลักประกันสุขภาพของท่านคืออะไร<br>ข้อเสนอแนะ:	☐ 0 ไม่มี<br>☐ 1 หลักประกันสุขภาพถ้วนหน้า<br>☐ 2 ประกันสังคม<br>☐ 3 กองทุนเงินทดแทนคนงาน<br>☐ 4 การเบิกค่ารักษาพยาบาลของข้าราชการ<br>☐ 5 อื่นๆ (ระบุ):
E.9	หลักประกันสุขภาพของบุตร/หลานของท่านคืออะไร	☐ 0 ไม่มี<br>☐ 1 หลักประกันสุขภาพถ้วนหน้า<br>☐ 2 ประกันสังคม<br>☐ 3 กองทุนเงินทดแทนคนงาน<br>☐ 4 การเบิกค่ารักษาพยาบาลของข้าราชการ<br>☐ 5 อื่นๆ (ระบุ):
E.10	ท่านจ่ายค่ายาต้านไวรัสของบุตร/หลานของท่านหรือไม่	☐ 1 ใช่<br>☐ 2 ไม่

หมวด F: รูปแบบการดำเนินชีวิต

	หัวข้อนี้เป็นส่วนสุดท้ายของการสัมภาษณ์<br>ทางโครงการจะถามท่านเล็กน้อยเกี่ยวกับรูปแบบการดำเนินชีวิตของท่านและถามว่าท่านเข้าใจเด็กที่ท่านดูแลแค่ไหน		
F.1	ท่านเป็นสมาชิกของกลุ่มที่ให้การสนับสนุนกลุ่มใดซึ่งสนับสนุนผู้ที่อาศัยอยู่ร่วมกับเอช ไอ วี หรือผู้ดูแลเด็กที่ติดเชื้อเอช ไอ วีหรือไม่	☐ 1 ใช่ ระบุ<br>☐ 2 ไม่	
F.2	ท่านนับถือศาสนาหรือไม่	☐ 1 ใช่<br>☐ 2 ไม่	⇒**Q** F.4
F.3	ถ้าใช่ ท่านนับถือศาสนาอะไร	☐ 1 พุทธ<br>☐ 2 คริสต์<br>☐ 3 อิสลาม<br>☐ 4 อื่นๆ (ระบุ:)	
F.4	ท่านคิดว่าสถานการณ์ด้านการเงินในปัจจุบันของท่านเป็นอย่างไร<br>ใช้ "*SMILE*" ***SCALE***<br>ข้อเสนอแนะ:	☐ 1 ลำบากมาก<br>☐ 2 ลำบาก<br>☐ 3 ธรรมดา<br>☐ 4 ดี<br>☐ 5 ดีมาก	
F.5	ในปัจจุบันท่านคิดว่าท่านมีความสุขแค่ไหน<br>ใช้ "*SMILE*" ***SCALE***<br>ข้อเสนอแนะ:	☐ 1 ไม่มีความสุขเลย<br>☐ 2 ไม่มีความสุข<br>☐ 3 ปานกลาง<br>☐ 4 มีความสุข<br>☐ 5 มีความสุขมาก	
F.5b	ท่านให้คะแนนความสามารถด้านสติปัญญาของเด็กแค่ไหน<br>ใช้ "*SMILE*" ***SCALE***<br>ข้อคิดเห็น: ....<br>...	☐ 1 ต่ำมาก<br>☐ 2 ต่ำ<br>☐ 3 ปานกลาง<br>☐ 4 ดี<br>☐ 5 ดีมาก	
F.6	ท่านคิดว่าเด็กมีความสุขแค่ไหน<br>ใช้ "*SMILE*" ***SCALE***<br>ข้อคิดเห็น: ....<br>...	☐ 1 ไม่มีความสุขเลย<br>☐ 2 ไม่มีความสุข<br>☐ 3 ปานกลาง<br>☐ 4 มีความสุข<br>☐ 5 มีความสุขมาก	

F.7	ท่านคิดว่าเด็ก<br>ใช้ *"SMILE" **SCALE***<br>ข้อคิดเห็น: ....<br>..	☐ 1 ดื้อมาก<br>☐ 2 ดื้อ<br>☐ 3 ธรรมดา<br>☐ 4 ดี<br>☐ 5 ดีมาก	
F.9	ท่านประเมินด้านการสื่อสารระหว่างท่านกับเด็กเป็นอย่างไร<br>ใช้ *"SMILE" **SCALE***<br>ข้อคิดเห็น: ....<br>..	☐ 1 ยากมาก<br>☐ 2 ยาก<br>☐ 3 ปานกลาง<br>☐ 4 ง่าย<br>☐ 5 ง่ายมาก	
F.8	เด็กช่วยท่านทำงานบ้านหรือไม่<br>(ตอบได้มากกว่าหนึ่งข้อ)<br>ข้อคิดเห็น: ....<br>..<br>..	☐ 1 ไม่	☐ 2 ใช่ ระบุ<br>☐ 1 ทำความสะอาดบ้าน<br>☐ 2 ซื้อของ<br>☐ 3 ทำอาหาร<br>☐ 4 ดูแลญาติที่อายุน้อยกว่า<br>☐ 5 ล้างจาน<br>☐ 6 ซักรีดเสื้อผ้า<br>☐ 7 ช่วยพ่อแม่ทำงานต่างๆ (งานในร้าน ในไร่ อื่นๆ)<br>☐ 8 อื่นๆ (ระบุ):
F.10	ท่านมีความขัดแย้งกับเด็กหรือไม่<br>ข้อคิดเห็น: ....<br>..	☐ 1 ตลอดเวลา<br>☐ 2 บางครั้ง<br>☐ 3 น้อยมาก<br>☐ 4 ไม่เคย	
F.11	ในกรณีที่มีความขัดแย้ง อะไรคือสาเหตุหลัก ....<br>....		
F.13	ท่านมีข้อเสนอแนะเกี่ยวกับแบบสอบถามนี้หรือไม่: ....<br>....		

ขอขอบคุณเป็นอย่างสูงสำหรับความร่วมมือของคุณ แม้คุณจะรู้สึกว่าบางคำถามค่อนข้างเป็นเรื่องส่วนตัว แต่คุณควรทราบไว้ว่าคำตอบของคุณนั้นสำคัญต่อการทำความเข้าใจสถานการณ์ของวัยรุ่นผู้ติดเชื้อเอช ไอ วี ในประเทศไทยให้ดีขึ้น และสำคัญต่อการกำหนดโครงการเพื่อพัฒนาสถานการณ์ของผู้ติดเชื้อเอช ไอ วี ในระดับชาติต่อไป

I. ความเห็นของผู้สัมภาษณ์

	เวลาสิ้นสุดการบันทึกข้อมูล: |___|___| นาฬิกา |___|___| นาที		
I.1	การสัมภาษณ์ดำเนินไปอย่างไร	☐ 1 ราบรื่น ไม่มีการหยุดชะงักใดๆ<br>☐ 2 ผู้ให้สัมภาษณ์แสดงอาการว่า อยากจะยุติการสัมภาษณ์<br>☐ 3 มีการหยุดชะงักหลายครั้ง	
I.2	อธิบายเกี่ยวกับผู้ให้สัมภาษณ์:<br>*(สามารถเลือกตอบได้มากกว่าหนึ่งคำตอบ)*	☐ 1 ผ่อนคลาย<br>☐ 2 ปานกลาง<br>☐ 3 มีความรู้สึกทางอารมณ์ง่าย<br>☐ 4 ดูนาฬิกาตลอดเวลา<br>☐ 5 มีความลำบากในการเข้าใจคำถาม<br>☐ 6 มีความลำบากในการแสดงความคิดออกมาเป็นคำ<br>☐ 7 มีความลำบากในการจำ หรือระบุวันที่เกิดเหตุการณ์<br>☐ 8 ร่างกายอ่อนล้า	
I.3	คำตอบค่อนข้าง	☐ 1 เชื่อถือได้มาก<br>☐ 2 เชื่อถือได้<br>☐ 3 ไม่น่าเชื่อถือ<br>☐ 4 เชื่อถือไม่ได้เลย<br>☐ 98 ไม่ทราบ	
