# Supplementary material for: Factors influencing antiretroviral treatment suboptimal adherence among perinatally HIV-infected adolescents in Thailand
Source: PLoS One. 2017 Feb 16;12(2):e0172392. doi: 10.1371/journal.pone.0172392 (PMC5312953; doi:10.1371/journal.pone.0172392)
Supplement: S5 File — (PDF) [file pone.0172392.s005.pdf]

Year	Age	Parent's status	Caregivers	Residence	School/Work	Disease	Health status	Age
**1**	**2**	**3**	**4**	**5**	**6**	**7**	**8**	**2**
25_ _	0							0
25_ _	1							1
25_ _	2							2
25_ _	3							3
25_ _	4							4
25_ _	5							5
25_ _	6							6
25_ _	7							7
25_ _	8							8
25_ _	9							9
25_ _	10							10
25_ _	11							11
25_ _	12							12
25_ _	13							13
25_ _	14							14
25_ _	15							15
25_ _	16							16
25_ _	17							17
25_ _	18							18
25_ _	19							19
25_ _	20							20

Comments:
