# Supplementary material for: Factors influencing antiretroviral treatment suboptimal adherence among perinatally HIV-infected adolescents in Thailand
Source: PLoS One. 2017 Feb 16;12(2):e0172392. doi: 10.1371/journal.pone.0172392 (PMC5312953; doi:10.1371/journal.pone.0172392)
Supplement: S1 Table — (DOCX) [file pone.0172392.s006.docx]

Supplemental Table 1: Odds ratio and Wald score of suboptimal adherence predictors in univariable analysis

Demographic Information:

| Predictor (N) |  | Suboptimal adherence n/N (%) | Univariable analysis | | | |
| --- | --- | --- | --- | --- | --- | --- |
|  |  |  | OR^a^ (95% CI) | P | Wald | P |
| Adolescent gender (568) | Male | 113/237 (47.7) | 1 |  | 0.088 | 0.766 |
|  | Female | 162/331 (48.9) | 1.052 (0.753-1.469) | 0.766 |  |  |
| Adolescent age (years) (568) | 12-13 | 123/249 (49.4) | 1 |  | 0.262 | 0.877 |
|  | 14-15 | 83/177 (46.9) | 0.905 (0.614-1.330) | 0.610 |  |  |
|  | 16-19 | 69/142 (48.6) | 0.968 (0.641-1.463) | 0.968 |  |  |
| Puberty (566) | No puberty | 96/189 (50.8) | 1 |  | 0.553 | 0.457 |
|  | Puberty | 179/377 (47.5) | 0.876 (0.617-1.242) | 0.457 |  |  |
| Intellectual ability assessed by caregiver (547) | Good, very good | 109/259 (42.1) | 1 |  | 9.839 | 0.007 |
|  | Average | 116/228 (50.9) | 1.425 (0.997-2.041) | 0.052 |  |  |
|  | Poor, very poor | 38/60 (63.3) | 2.377 (1.341-4.301) | 0.003 |  |  |
| Caregiver type (568) | Parents | 69/179 (38.5) | 1 |  | 12.561 | 0.002 |
|  | Grandparents | 105/213 (49.3) | 1.550 (1.037-2.325) | 0.033 |  |  |
|  | Other family members | 101/176 (57.4) | 2.147 (1.408-3.291) | <0.001 |  |  |
| Caregiver gender (568) | Male | 57/127 (44.9) | 1 |  | 0.817 | 0.366 |
|  | Female | 218/441 (49.4) | 1.201 (0.808-1.789) | 0.366 |  |  |
| Caregiver age (years) (568) | ≤50 | 133/292 (45.4) | 1 |  | 1.976 | 0.160 |
|  | >50 | 142/276 (51.4) | 1.267(0.911-1.764) | 0.160 |  |  |
| Caregiver education (568) | None | 36/83 (43.4) | 1 |  | 1.012 | 0.603 |
|  | Primary | 187/378 (49.5) | 1.278 (0.794-2.073) | 0.315 |  |  |
|  | Secondary and above | 52/107 (48.6) | 1.234 (0.694-2.203) | 0.474 |  |  |
| Age of ART Initiation (years) (568) | <10 | 174/353 (49.3) | 1 |  | 0.287 | 0.592 |
|  | ≥10 | 101/215 (47.0) | 0.911 (0.649-1.280) | 0.592 |  |  |
| Years on ART (568) | 0-4 | 104/216 (48.1) | 1 |  | 1.307 | 0.520 |
|  | 5-6 | 86/188 (45.7) | 0.908 (0.613-1.343) | 0.629 |  |  |
|  | ≥7 | 85/164 (51.8) | 1.159 (0.772-1.741) | 0.477 |  |  |
| ART treatment (568) | NNRTI based | 178/420 (42.4) | 1 |  | 22.754 | <0.001 |
|  | Other | 97/148 (65.5) | 2.586 (1.757-3.841) | <0.001 |  |  |
| Medicine side effects reported by adolescent (568) | No | 236/494 (47.8) | 1 |  | 0.625 | 0.429 |
|  | Yes | 39/74 (52.7) | 1.218 (0.747-1.994) | 0.429 |  |  |
| Disclosure of HIV status reported by caregiver (568) | Adolescent does not know | 30/66 (45.5) | 1 |  | 0.278 | 0.870 |
|  | Formal disclosure | 170/347 (49.0) | 1.153 (0.680-1.964) | 0.441 |  |  |
|  | Adolescent knows without formal disclosure | 75/155 (48.4) | 1.125 (0.632-2.013) | 0.690 |  |  |
|  |  |  |  |  |  |  |

Adolescent self-administered questionnaire:

| Predictor (N) |  | Suboptimal adherence n/N (%) | | Univariable analysis | | | |
| --- | --- | --- | --- | --- | --- | --- | --- |
|  |  |  |  | OR (95% CI) | P | Wald | P |
| **School** | | | | | | | |
| School attendance (568) | No | 55/100 (55.0) | | 1 |  | 2.097 | 0.148 |
|  | Yes | 220/468 (47.0) | | 0.726 (0.469-1.119) | 0.148 |  |  |
| Extra-curriculum attendance (566) | No | 138/263 (52.5) | | 1 |  | 3.239 | 0.072 |
|  | Yes | 136/303 (44.9) | | 0.738 (0.529-1.027) | 0.072 |  |  |
| Work (568) | No | 161/356 (45.2) | | 1 |  | 3.877 | 0.049 |
|  | Yes | 114/212 (53.8) | | 1.409 (1.002-1.984) | 0.049 |  |  |
| Type of student (563) | Good, very good | 113/255 (44.3) | | 1 |  | 3.249 | 0.071 |
|  | Fair, bad, very bad | 140/308 (51.9) | | 1.359 (0.974-1.898) | 0.071 |  |  |
| Plan to attend college (568) | Yes | 129/283 (45.6) | | 1 |  | 1.810 | 0.179 |
|  | No or don’t know | 146/285 (51.2) | | 1.254 (0.902-1.745) | 0.179 |  |  |
| **Relationship with caregivers and health providers** | | | | | | | |
| Arguments with caregiver (567) | Never or rarely | 119/267 (44.6) | | 1 |  | 5.988 | 0.050 |
|  | Sometimes | 103/213 (48.4) | | 1.165(0.812-1.672) | 0.408 |  |  |
|  | Often or very often | 52/87 (59.8) | | 1.848 (1.134-3.040) | 0.014 |  |  |
| Easiness to ask caregiver questions (541) | Very easy | 77/168 (45.8) | | 1 |  | 6.912 | 0.075 |
|  | Easy | 93/201 (46.3) | | 1.018 (0.675-1.536) | 0.933 |  |  |
|  | Ok | 73/148 (49.3) | | 1.150 (0.739-1.793) | 0.535 |  |  |
|  | Difficult or very difficult | 18/24 (75.0) | | 3.545 (1.409-10.181) | 0.011 |  |  |
| Easiness to ask doctors questions (567) | Very easy | 76/134 (56.7) | | 1 |  | 7.488 | 0.058 |
|  | Easy | 94/204 (46.1) | | 0.652 (0.419-1.010) | 0.056 |  |  |
|  | Ok | 92/210 (43.8) | | 0.595 (0.383-0.920) | 0.020 |  |  |
|  | Very difficult, difficult | 12/19 (63.2) | | 1.308 (0.494-3.709) | 0.596 |  |  |
| **Social life** | | | | | | | |
| Happiness (568) | Very happy | 34/98 (34.7) | 1 | |  | 11.944 | 0.008 |
|  | Happy | 94/189 (49.7) | 1.863 (1.130-3.106) | | 0.016 |  |  |
|  | Average | 84/174 (48.3) | 1.757 (1.059-2.949) | | 0.031 |  |  |
|  | Unhappy or very unhappy | 63/107 (58.9) | 2.695 (1.538-4.788) | | 0.001 |  |  |
| Boy/girlfriend (567) | No | 199/441 (45.1) | 1 | |  | 8.919 | 0.003 |
|  | Yes | 76/126 (60.3) | 1.848 (1.239-2.778) | | 0.003 |  |  |
| Hang out with friends (560) | No | 105/220 (47.7) | 1 | |  | 1.508 | 0.470 |
|  | Sometimes | 140/291 (48.1) | 1.015 (0.715-1.442) | | 0.932 |  |  |
|  | Often | 28/49 (57.1) | 1.460 (0.785-2.753) | | 0.235 |  |  |
| Has ever been drunk (522) | No | 191/425 (44.9) | 1 | |  | 6.870 | 0.009 |
|  | Yes | 58/97 (59.8) | 1.822 (1.167-2.870) | | 0.009 |  |  |
| Ride motorbike fast (568) | Don’t know how to ride | 45/96 (46.9) | 1 | |  | 0.111 | 0.946 |
|  | No | 147/302 (48.7) | 1.075 (0.679-1.706) | | 0.758 |  |  |
|  | Yes | 83/170 (48.8) | 1.081 (0.655-1.788) | | 0.760 |  |  |
| Ever had motorbike accident (567) | No | 194/408 (47.5) | 1 | |  | 0.350 | 0.554 |
|  | Yes | 80/159 (50.3) | 1.117 (0.774-1.673) | | 0.554 |  |  |
| Know people using drugs (566) | No | 208/441 (47.2) | 1 | |  | 1.610 | 0.205 |
|  | Yes | 67/125 (53.6) | 1.294 (0.870-1.930) | | 0.205 |  |  |
| Play computer games (568) | No | 50/117 (42.7) | 1 | |  | 2.925 | 0.232 |
|  | Sometimes | 182/373 (48.8) | 1.277 (0.841-1.947) | | 0.253 |  |  |
|  | Regularly | 43/78 (55.1) | 1.646 (0.926-2.945) | | 0.091 |  |  |
| Chat on internet (567) | No | 127/287 (44.3) | 1 | |  | 5.711 | 0.058 |
|  | Sometimes | 129/237 (54.4) | 1.505 (1.066-2.129) | | 0.021 |  |  |
|  | Often | 19/43 (44.2) | 0.997 (0.518-1.897) | | 0.994 |  |  |

Caregiver Interviews:

| Predictor (N) |  | Suboptimal adherence n/N (%) | Univariable analysis | | | |
| --- | --- | --- | --- | --- | --- | --- |
|  |  |  | OR (95% CI) | P | Wald | P |
| **Household situation** | | | | | | |
| Financial situation (568) | Ok, good, very good | 179/360 (49.7) | 1 |  | 0.672 | 0.412 |
|  | Very difficult, difficult | 96/208 (46.2) | 0.867 (0.615-1.220) | 0.412 |  |  |
| Conflict with adolescent (568) | Never or rarely | 162/334 (48.5) | 1 |  | 0.002 | 0.960 |
|  | Sometimes, all the time | 113/234 (48.3) | 0.992 (0.710-1.385) | 0.960 |  |  |
| Communication with adolescent (568) | Easy, very easy | 197/426 (46.2) | 1 |  | 3.203 | 0.074 |
|  | Ok, difficult, or very difficult | 78/142 (54.9) | 1.417 (0.968-2.079) | 0.074 |  |  |
| Adolescent obedience (567) | Nice, very nice | 128/293 (43.7) | 1 |  | 5.208 | 0.022 |
|  | Average, somehow or very disobedient | 146/274 (53.3) | 1.470 (1.057-2.050) | 0.022 |  |  |
| **HIV-related discrimination** | | | | | | |
| People in the community know HIV status (568) | No or unclear | 60/125 (48.0) | 1 |  | 0.011 | 0.916 |
|  | Yes | 215/443 (48.5) | 1.022 (0.687-1.522) | 0.916 |  |  |
| School staff know HIV status (566) | No or unclear | 85/183 (46.4) | 1 |  | 0.345 | 0.557 |
|  | Yes | 188/383 (49.1) | 1.112 (0.781-1.583) | 0.557 |  |  |
| Students at school know HIV status (565) | No or unclear | 106/236 (44.9) | 1 |  | 1.688 | 0.194 |
|  | Yes | 166/329 (50.5) | 1.249 (0.894-1.748) | 0.194 |  |  |
| Discrimination from family (568) | No or unclear | 261/545 (47.9) | 1 |  | 1.459 | 0.227 |
|  | Yes | 14/23 (60.9) | 1.693 (0.730-4.125) | 0.227 |  |  |
| Discrimination from friends (568) | No or unclear | 203/442 (45.9) | 1 |  | 4.898 | 0.027 |
|  | Yes | 72/126 (57.1) | 1.570 (1.055-2.348) | 0.027 |  |  |
| Discrimination at school (568) | No or unclear | 203/433 (46.9) | 1 |  | 1.711 | 0.191 |
|  | Yes | 72/135 (53.3) | 1.295 (0.880-1.910) | 0.191 |  |  |
| Discrimination in the neighborhood (568) | No or unclear | 231/476 (48.5) | 1 |  | 0.015 | 0.902 |
|  | Yes | 44/92 (47.8) | 0.972 (0.621-1.520) | 0.902 |  |  |

^a^ OR=Odds ratio
